# Mutational Characteristics of Causative Genes in Chinese Hereditary Spherocytosis Patients: a Report on Fourteen Cases and a Review of the Literature

**DOI:** 10.3389/fphar.2021.644352

**Published:** 2021-07-16

**Authors:** Dong Wang, Li Song, Li Shen, Kaihui Zhang, Yuqiang Lv, Min Gao, Jian Ma, Ya Wan, Zhongtao Gai, Yi Liu

**Affiliations:** ^1^ Pediatric Research Institute, Qilu Children’s Hospital of Shandong University, Jinan, China; ^2^ Pediatric Hematology and Oncology, Qilu Children’s Hospital of Shandong University, Jinan, China; ^3^ Clinical Laboratory, The Fourth Hospital of Jinan, Jinan, China

**Keywords:** hereditary spherocytosis, mutation, ANK1, SPTB, whole exome sequencing

## Abstract

**Background:** Hereditary spherocytosis (HS), characterized by the presence of spherocytic red cells in peripheral blood, hemolysis, splenomegaly, jaundice, and gallstones, is a common form of inherited hemolytic anemia (HA). To date, five causative genes associated with HS have been identified, including *ANK1, SPTB, SPTA1, SLC4A1*, and *EPB42*.

**Methods:** Clinically suspected patients with HS or undiagnosed HA from 14 Chinese families were enrolled in this study. We presented the patients’ clinical features and identified the causative gene variants in these patients using whole exome sequencing (WES), with 10 novel and four reported mutations in the *ANK1* and *SPTB* genes (seven mutations in *ANK1* and seven in *SPTB*), individually. Then, we reviewed all available literature on Chinese HS patients from 2000 to 2020 in PubMed and Chinese Journals with genetic results and clinical information, to delineate gene mutation spectrum and potential correlation with phenotypes.

**Results:** A total of 158 variants (including 144 in previous reports and 14 in this study) indicated that *ANK1* (46%) and *SPTB* (42%) were the most frequently mutated genes in Chinese HS patients, followed by *SLC4A1* (11%) *and SPTA1* (1%), while no mutations in *EPB42* was reported. Most of the mutations in *ANK1* and *SPTB* were nonsense (26/73 in *ANK1* and 32/66 in *SPTB*) and frameshift (20/73 in *ANK1* and 15/66 in *SPTB*), while missense mutations (14/18) accounted for the majority in *SLC4A1*. The higher mutation frequency of *ANK1* was found in its exon 8, 9, 26, and 28. The majority of mutations in *SPTB* were located in its exon 13, 15, and 18–30, whereas mutations in *SLC4A1* were scattered throughout the entire region of the gene.

**Conclusion:** Our study expanded the mutation spectrum of *ANK1* and *SPTB*. Furthermore, we clarified the mutational characteristics of causative genes by reviewing all available literature on Chinese patients with HS.

## Introduction

Hereditary spherocytosis (HS) refers to a group of heterogeneous disorders, and is a common form of congenital hemolytic anemia (HA). It is characterized by the presence of sphere-shaped red blood cells (spherocytes) on the peripheral blood smear, anemia, jaundice, and splenomegaly, with wide heterogeneity in severity ranging from virtually asymptomatic conditions to severe forms that require transfusions in early childhood, which can make an exact diagnosis of HS cases quite difficult, particularly for asymptomatic or atypical cases if only depending on clinical manifestations, family history and hematologic laboratory tests (Bolton-Maggs et al., 2012). It has been reported worldwide with a prevalence that varies from 1:2,000 in Caucasians to 1:100,000 in Chinese cases (Perrotta et al., 2008; Wang et al., 2018). The study of the gene mutation spectrum has been subject to some general concerns.

Early diagnosis of HS is necessary to prevent adverse outcomes, and if diagnosed too late, HS cases are prone to risk of long-term complications, such as cholelithiasis, hemolytic episodes, and aplastic crises; furthermore, non-diagnosed HS may lead to a severe neurological complication called kernicterus (Perrotta et al., 2008; Will et al., 2017; Ciepiela, 2018; Brosius, 2019). Splenectomy is curative and considered the standard surgical treatment for those with moderate to severe HS conditions but has a life-long risk of potentially lethal infections (Manciu et al., 2017). Since traditional laboratory tests often fail to diagnose HS, particularly asymptomatic or atypical cases, molecular genetic testing, especially next-generation sequencing is becoming a powerful tool for clinical diagnosis of HS in neonates or infants with its capability of accurately identifying genetic variants (Long and Shen, 2019; Shen and Shi, 2019; Yang et al., 2019).

The molecular basis of HS is due to defects in red cell membrane proteins which result in decreased membrane surface area and resultant deformability of the erythrocytes, thereby accelerating the degradation and hemolysis in the spleen. The severity of the anemia is directly associated with the extent of membrane surface area loss and consequent increase of cell sphericity (Narla and Mohandas, 2017; Iolascon et al., 2019). The deficiency of membrane in the erythrocytes is caused by their corresponding gene mutations, including *ANK1*, *SPTA1*, *SPTB*, *SLC4A1,* and *EPB42*, which encodes ankyrin 1, spectrin *a*-chain, spectrin *ß*-chain, anion exchanger 1 (band 3), and protein 4.2, respectively (Perrotta et al., 2008; Da Costa et al., 2013; Narla and Mohandas, 2017; Iolascon et al., 2019). Autosomal dominant inheritance is the main inherited manner of HS accounting for 75%, while autosomal recessive (AR) and non-dominant inheritance have also been described in about 25% of cases (Delaunay, 1995; Iolascon and Avvisati, 2008; Garcon, 2009; Zamora and Schaefer, 2019). Thus, the definitive diagnosis of HS is determined by molecular diagnostic study. It has been reported that the mutation in *ANK1* (∼50%) and spectrin gene (*SPTB*: ∼20% and *SPTA1*: ∼5%) is the major cause of HS, followed by a mutation in *SLC4A1* (∼15%) and *EPB42* (∼10%) (An and Mohandas, 2008; Zamora and Schaefer, 2019). Until recently, next-generation sequencing was performed on patients with HS from different countries and regions, such as America, Europe, Brazil, and Korea (Agarwal et al., 2016; Mansour-Hendili et al., 2020; Svidnicki et al., 2020; Shin et al., 2018). There have been some case reports in Chinese HS patients (Wang et al., 2018; Meng et al., 2019; Qin et al., 2020). However, the gene mutation spectrum has not been delineated well to date.

In the present study, we investigated causative gene mutations in a cohort of 14 patients with clinically suspected HS or undiagnosed HA from Shandong Province, a northern area of China, using next generation sequencing. The 14 patients were from 14 unrelated families, including one neonate, six infants, and seven children over one-year-old. We identified 10 novel mutations in ANK1 and *SPTB* genes. In addition, we discussed the clinical features of these patients in the light of the previous reports regarding mutations of the causative genes in Chinese HS patients to provide information for genetic counseling, prenatal screening, and future research of individual therapy.

## Materials and Methods

### Ethics Statement

This work was approved by the Medical Ethics Committee of Qilu Children’s Hospital of Shandong University. Clinical and laboratory examinations were performed on the probands and their parents after informed consent was obtained. The information of all patients was anonymized before submission. All the procedures performed in this work were in accordance with the Helsinki Declaration.

### Patients

Fourteen hospitalized children (8 boys and six girls) from 14 unrelated Chinese families with clinically suspected HS or undiagnosed HA were recruited for this study in Qilu Children’s Hospital, Shandong University (QCHSU) from February 2016 to July 2020. All probands from the Han Chinese population in Shandong Province, China were examined and diagnosed by experienced hematology specialists from the Hematology or Hematology Oncology Department of QCHSU (Table 1).

**TABLE 1:** The clinical and laboratory features of the Chinese patients with HS.

Patients		P 1	P 2	P 3	P 4	P 5	P 6	P 7	P 8	P9	P10	P11	P12	P13	P14	
Gender		M	F	M	M	M	M	M	F	F	M	M	F	F	F	
Age		1 y4 m	1 m7 d	1 m1 d	11 m4 d	1 y3 m	8 d	11 y	5 m20 d	1 y1 m	1 m18 d	7 y	1 m6 d	1 y5 m	6 y	
Age at the onset		2 m	3 d	1 d	1 d	NA	1 d	6 y	2 d	1 d	2 d	7 y	2 d	1 y4 m	15 d	
Clinical symptoms	Jaundice	−	+	+	+	+	+	+	+	+	+	−	+	+	+
	Anemia	+	+	+	+	+	+	+	+	+	+	+	+	+	+
	Splenomegaly	+	−	−	−	NA	−	+	+	+	+	−	−	−	+
Lab tests	RBC (×10^12^/L)	2.12	1.95	1.92	1.85	3.18	3.19	2.28	1.98	2.61	1.79	1.95	1.66	3.78	2.48
	Hemoglobin (g/L)	50	60	59	56	92	109	79	57	64	54	54	54	109	76
	MCV (fL)	72.4	86.2	84.2	83.8	81.8	96.6	99.8	82.8	77.8	91.1	77.4	94.1	86.8	98.0
	MCH (pg)	23.4	30.8	29.2	28.9	28.9	34.2	34.7	29.0	24.5	30.2	27.8	32.3	28.8	30.6
	MCHC (g/L)	323	357	347	346	354	354	347	350	315	338	359	343	332	313
	Reticulocytes (%)	4.10	3.56	7.63	5.78	12.92	8.44	14.23	8.47	12.20	6.30	14.40	8.88	11.70	17.02
	Total bilirubin (μmol/L)	22.0	484.7	202.0	272.3	54.4	510.8	87.7	395	38	179	34.1	146.4	148.9	55.7
	LDH (U/L)	349	384	234	471	354	572	401	757	324	541	424	233	239	399
	Spherocytes on the peripheral blood smears	−	+	+	+	+	+	+	+	+	+	+	+	+	+
	Direct antiglobulin test	−	−	−	−	−	−	−	−	−	−	−	−	−	−
	Increased osmotic fragility	−	−	−	−	+	+	+	−	+	NA	NA	+	+	+
Family history		HA	HS	HS	−	−	−	−	HS	−	−	HA	HS	−	−	
Clinical diagnosis		HA	HS	HS	HS	HS	HS	HS	HS	HS	HS	HS	HS	HS	HS	

M, male; F, female; HA, hemolytic anemia; HS, Hereditary spherocytosis; LDH, lactate dehydrogenase; MCH, mean corpuscular hemoglobin; MCHC, mean corpuscular hemoglobin concentration; MCV, mean corpuscular volume; RBC, red blood cell. NA: not available; y, year(s); m, month(s); d, day(s); “+”: Present; “−”: Absent.

### Genetic Analysis

Blood samples collected in EDTA vacutainer were obtained from the probands and their parents for DNA extraction using QIAamp DNA Blood Midi Kit (Qiagen, Shanghai, China) according to the manufacturer’s protocol. Whole exome sequencing (WES) with the Human Exome Probes P039-Exome (MyGenostics, Beijing, China) on the Illumina NovaSeq5000 platform (Illumina, United States) was applied for the mutation screening of the probands. The obtained mean exome coverage was more than 95% (>10X coverage; mean depth of over 100X). Paired-end alignment was performed using Burrows-Wheeler Aligner software (BWA Version: 0.7.10) to version GRCh37/hg19 of the human genome. SAM files then were sorted and converted to BAM. BAM files were filtered and duplicates were marked with Picard. Local realignment was performed using GATK’s RealignerTargetcreator and IndelRealigner, base quality score recalibration using GATK’s BaseRecalibrator, and variants were called jointly in all samples using the GATK’s HaplotypeCaller in the “GENOTYPE_GIVEN_ALLELES” mode. Then, GATK’s VariantFiltration was used for filtering SNPs and Indels. ANNOVAR was used for annotation (http://wannovar.wglab.org/) after variant detection. Variant frequencies were determined in thousands of genomes (http://www.1000genomes.org), ExAC (http://exac.broadinstitute.org/), Exome Variant Server (EVS,http://evs.gs.washington.edu/EVS) and in-house database to remove common variants (suballelic frequency >5%). Then, the variants resulting in frameshift, missense variants, premature stop-gain, or initiation codon loss, and typical splicing site changes were prioritized for study. SIFT, PolyPhen-2, MutationTaster, and REVEL were used to evaluate the novel variants’ pathogenicity. Moreover, genetic variations included in HGMD (http://www.hgmd.cf.ac.uk) and ClinVar (http://www.ncbi.nlm.nih.gov/clinvar) will also be further analyzed. The variants identified in this study were classified according to the 2015 American College of Medical Genetics and Genomics (ACMG) guidelines (Richards et al., 2015).

### Validation of Gene Mutations

Sanger sequencing was then utilized to validate the definitely and likely pathogenic variants identified by whole exome sequencing in the patients with designed specific primers. Sanger validation primer sets were designed using Primer Premier v5.0 software. PCR amplification was performed using AmpliTaq Gold^®^ 360 DNA polymerase (Applied Biosystems). PCR products were further purified and sequenced using an ABI Prism 3700 automated sequencer (Applied Biosystems, Foster City, CA).

### Literature Review and Statistical Analysis

To delineate gene mutation spectrum and potential correlation with phenotypes, we reviewed all available published reports of Chinese HS patients from 2000 to 2020 in PubMed and Chinese Journals and classified all the cases into different groups based on 1) mutated genes: *AN*K1 group, *SPTB* group, and *SLC4*A1 group; 2) types of mutations: missense group, nonsense group, frameshift group, and splicing group. The genotype-phenotype correlation was analyzed by comparing the clinical parameters of the patients, such as hemoglobin, total bilirubin, and reticulocytes among different groups. The association analysis was determined using the Kruskal–Wallis test. The significance of association was estimated by calculating the odds ratio and relative risk with a 95% confidence interval (CI). All tests were two tailed, and *p*-value < 0.05 was considered statistically significant. All statistical analyses were performed using SPSS v19.0 software.

## Results

### Clinical Manifestations and Laboratory Tests

All patients age from 8 days to 11 years old manifesting anemia and jaundice (except for P1 and P11) with varying severity. Of them, eight patients (P2, P3, P4, P6, P8, P9, P12, and P14) presented anemia or jaundice in the neonatal period, and six patients (P1, P7, P8, P9, P10, and P14) displayed splenomegaly on abdominal ultrasound. Blood biochemical index showed variable low level hemoglobin (Hb), reticulocytosis, and hyperbilirubinemia (unconjugated). Anisocytosis or spherical red blood cells were observed on the peripheral blood smear of all the patients (except for P1). Autoimmune hemolytic anemia was excluded by a direct antiglobulin test. Seven of the patients presented an increase in erythrocyte osmotic fragility. Moreover, six patients had a family history of HS or HA. Clinical data from them are shown in Table 1.

### Gene Mutation Test

In total, 14 different heterozygous candidate mutations were identified including 10 novel and four reported, with seven in *ANK1* and seven in *SPTB*, nine nonsense, and five frameshift mutations (Table 2). All 10 novel mutations can be considered null mutations and result in a premature terminator codon in the coding sequence of *ANK1* or *SPTB* that may cause a defective protein product lacking key domains of ankyrin one or *ß*-spectrin. The validation of parental genetic tests showed that seven mutations (5 in *ANK1* and two in *SPTB*) were *de novo* and the others were inherited mutations. All the 14 mutations were finally determined to be pathogenic after analyzing according to the latest ACMG guidelines (Table 2), all 14 probands were genetically confirmed as HS patients and interventions took place to prevent complications.

**TABLE 2:** Mutations analyzed by WES and validated by Sanger sequencing in the patients from eight Chinese families.

Patients	Clinical diagnosis	Gene	Exon	DNA change	Effect	Mutation type	dbSNP/1000G/EVS/ExAC	Status	Inheritance	Pathogenicity prediction score (Mutation Taster)	Pathogenic evaluation according to ACMG
**P1**	HA	*ANK1*	31	c.3754C > T	p.R1252*	Nonsense	0/0/0/0	Reported (Nakanishi et al., 2001)	Paternal	1	Pathogenic
**P2**	HS	*SPTB*	25	c.5266C > T	p.R1756*	Nonsense	0/0/0/0	Reported (Maciag et al., 2009)	Paternal	1	Pathogenic
**P3**	HS	*SPTB*	13	c.1912C > T	p.R638*	Nonsense	0/0/0/0	Reported Qin et al. (2020)	Maternal	1	Pathogenic
**P4**	HS	*SPTB*	15	c.3168dupG	p.L1057Afs*16	Frameshift	0/0/0/0	Novel	Paternal	1	Pathogenic
**P5**	HS	*SPTB*	24	c.4978C > T	p.Q1660*	Nonsense	0/0/0/0	Novel	*De novo*	1	Pathogenic
**P6**	HS	*ANK1*	4	c.319C > T	p.Q107*	Nonsense	0/0/0/0	Novel	*De novo*	1	Pathogenic
**P7**	HS	*SPTB*	27	c.5933_5934delAG	p.E1978G*18	Frameshift	0/0/0/0	Novel	*De novo*	1	Pathogenic
**P8**	HS	*ANK1*	7	c.709C > T	p.Q237*	Nonsense	0/0/0/0	Novel	Paternal	1	Pathogenic
**P9**	HS	*ANK1*	26	c.2950C > T	p.Q984*	Nonsense	0/0/0/0	Novel	*De novo*	1	Pathogenic
**P10**	HA	*ANK1*	31	c.3813_3823del	p.Q1272Lfs*100	Frameshift	0/0/0/0	Novel	*De novo*	1	Pathogenic
**P11**	HS	*SPTB*	18	c.3984G > A	p.W1328*	Nonsense	0/0/0/0	Novel	Maternal	1	Pathogenic
**P12**	HS	*SPTB*	15	c.3448dupT	p.W1150L*32	Frameshift	0/0/0/0	Novel	Paternal	1	Pathogenic
**P13**	HS	*ANK1*	9	c.856C > T	p.R286*	Nonsense	0/0/0/0	Reported Wang et al. (2018)	*De novo*	1	Pathogenic
**P14**	HS	*ANK1*	31	c.3847delA	p.R1283Gfs*3	Frameshift	0/0/0/0	Novel	*De novo*	1	Pathogenic

The variants are described using NM_020476.2 for *ANK1* and NM_001024858.2 for *SPTB* transcript reference sequences. *De novo:* build up from nothing.

### The Literature Review and Mutational Characteristics of Chinese HS Patients

**FIGURE 1:**
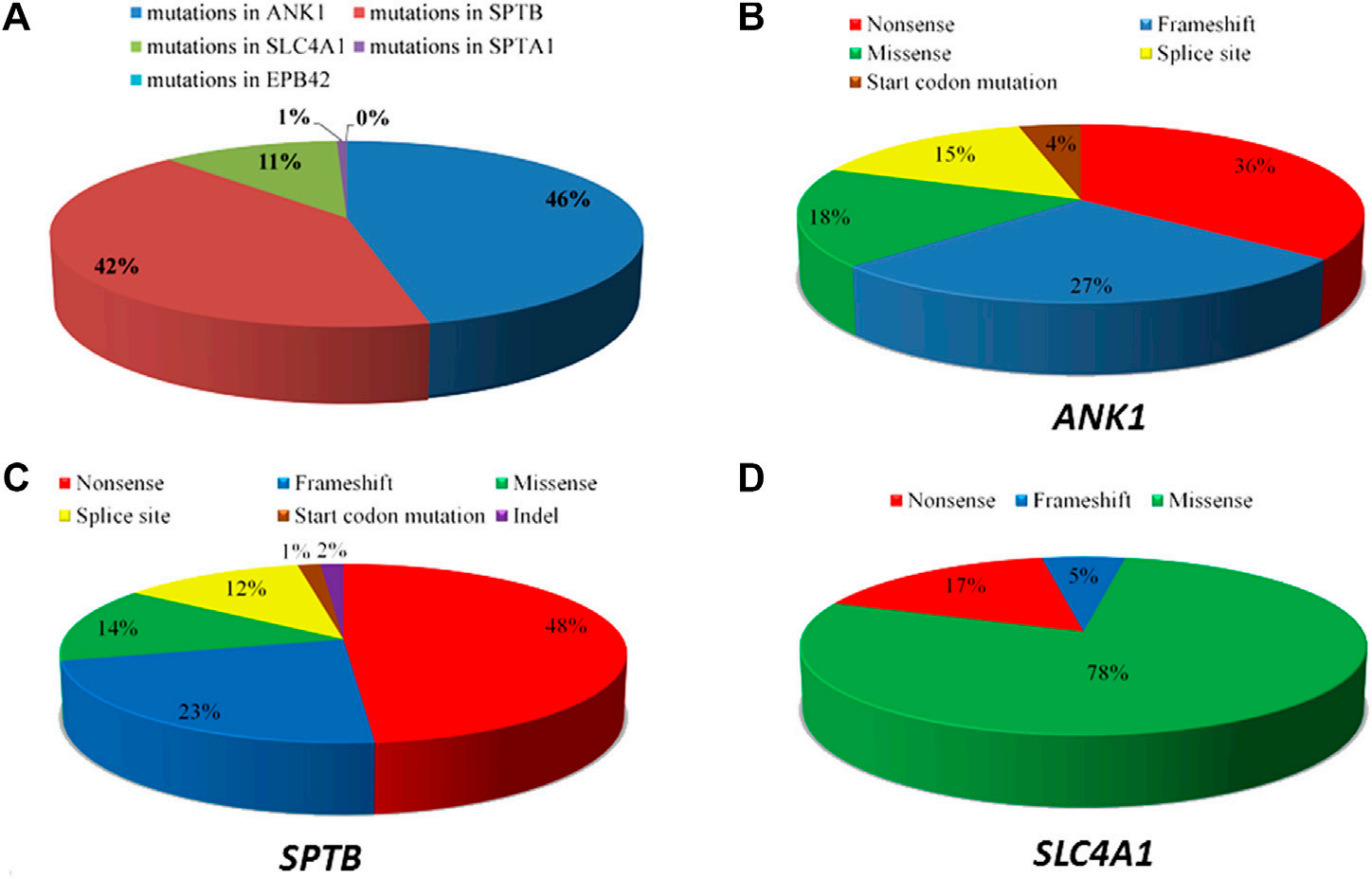
Summary of the causative genes spectrum of Chinese HS patients. **(A)** The distribution of five causative genes (*ANK1, SPTB, SPTA1, SLC4A1*, and *EPB42*) mutations. **(B)** Types of *ANK1* mutations. **(C)** Types of *SPTB* mutations. **(D)** Types of *SLC4A1* mutations.

**FIGURE 2:**
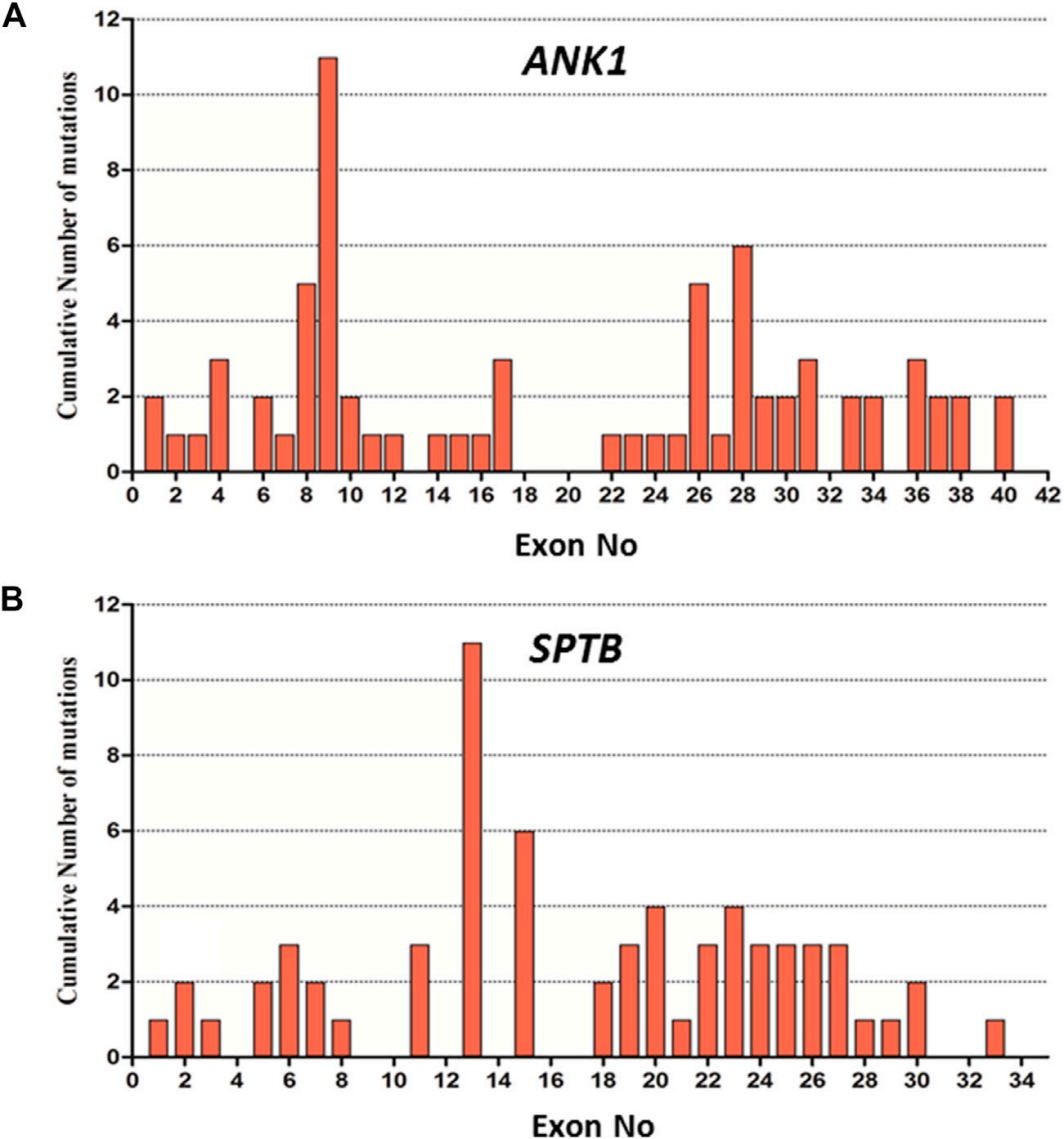
Distribution of mutations in the exon of *ANK1* and *SPTB* gene. **(A)** Cumulative Number of mutations in each exon of the *ANK1* gene. **(B)** Cumulative Number of mutations in each exon of *SPTB* gene.

We summarized data for 158 cases from 144 Chinese patients with HS (genetically confirmed cases) previously reported in the literature and 14 HS cases identified in this study (Supplementary Table 1), and the mutational characteristics of causative genes in all these patients were summarized. In total, 158 mutations consisted of 144 previous reports and 14 mutations in this study with 157 heterozygous in four genes of *ANK1, SPTB, SLC4A1,* and *SPTA1*, while one exception of homozygous in *SPTB*, including 73 (46%) in *ANK1*, 66 (42%) in *SPTB*, 18 (11%) in *SLC4A1*, 1 (1%) in *SPTA1,* but no *EPB42* (0%), which indicates that *ANK1* and *SPTB* mutations are two major causes of HS in the Chinese population. The types of gene mutations varied in the different genes, for example, the mutations in *ANK1* including 26 (36%) nonsense, 20 (27%) frameshift, 13 (18%) missense, 11 (15%) splicing and 3 (4%) nucleotide substitutions in the start codon. The mutations in *SPTB* included 32 (48%) nonsense, 15 (23%) frameshift, 9 (14%) missense, 8 (12%) splicing, 1 (1.5%) nucleotide substitution in start condon, and 1 (1.5%) indel mutations. The mutations in *SLC4A1* included 3 (17%) nonsense, 1 (5%) frameshift and 14 (78%) missense mutations. Therefore, the majority of mutation types in both *ANK1* and *SPTB* were nonsense (26/73 in *ANK1* and 32/66 in *SPTB*) followed by frameshift (20/73 in *ANK1* and 15/66 in *SPTB*), but missense mutations (14/18) accounted for the majority of the *SLC4A1* mutations. Figure 2 shows that exon 8, 9, 26, and 28 of the *ANK1* gene have a higher mutation frequency*,* and the majority of *SPTB* mutations were located in exon 13, 15, and 18–30, whereas *SLC4A1* mutations were scattered throughout the entire gene. The analysis of all the mutations is shown in Figure 1 and Figure 2.

### Genotype–Phenotype Correlation in HS Patients

To investigate the genotype–phenotype association of all HS patients, we first divided the patients into different groups based on the mutated genes and types of mutations, separately, then analyzed their data among the groups by Kruskal–Wallis test which showed no significant differences regarding Hb level, reticulocytes, and total bilirubin among different groups (Table 3, Table 4).

**TABLE 3:** Comparison of clinical features of HS patients with *SPTB*, *ANK1* and *SLC4A1* mutations.

Clinical data	*ANK1* (*n* = 73)	*SPTB* (*n* = 66)	*SLC4A1* (*n* = 18)	*p*-value
Hb (g/L), median (range)	72.4 (26.0–148.0) *n* = 34	76.3 (10.8–125) *n* = 33	96.0 (56.0–120.0) *n* = 4	0.131
Ret (%), median (range)	10.86 (3.09–23.57) *n* = 25	9.82 (1.10–17.44) *n* = 27	12.95 (5.60–20.30) *n* = 2	0.884
T-Bil (μmol/L), median (range)	125.9 (22.0–521.2) *n* = 26	112.0 (25.1–484.7) *n* = 26	125.4 (45.2–257.0) *n* = 3	0.768

*p*-values of <0.05 were considered statistically significant. Hb, hemoglobin; Ret, reticulocyte; T-Bil, total bilirubin.

**TABLE 4:** Comparison of clinical features of HS patients with different types of mutation.

Clinical data	Nonsense (*n* = 61)	Frameshift (*n* = 36)	Missense (*n* = 36)	Splicing (*n* = 19)	*p*-value
Hb (g/L), median (range)	74.2 (10.8–125) *n* = 28	76.2 (40.0–148.0) *n* = 19	80.7 (55.0–120.0) *n* = 13	73.5 (56.0–104.0) *n* = 10	0.853
Ret (%), median (range)	10.29 (3.56–17.88) *n* = 20	9.74 (1.93–23.57) *n* = 16	12.06 (1.10–20.30) *n* = 9	12.95 (5.60–20.30) *n* = 7	0.656
T-Bil (μmol/L), median (range)	144.4 (22.0–510.8) *n* = 22	122.5 (39.6–521.2) *n* = 16	72.1 (25.1–177.2) *n* = 8	93.5 (52.3–118.1) *n* = 8	0.385

*p*-values of <0.05 were considered statistically significant. Hb, hemoglobin; Ret, reticulocyte; T-Bil, total bilirubin.

## Discussion

The molecular basis of HS is the mutations in the genes coding red cell membrane components (Delaunay, 2007). Therefore, the detection of these mutations is the principal method to accurately diagnose HS. In the present study, 14 different heterozygous candidate mutations (10 novel and four reported) were identified in all 14 Chinese HS families, and seven families harbored *ANK1* mutations (7/14, 50%), and seven harbored *SPTB* mutations (7/14, 50%), whereas no mutation in the *SPTA1*, *SLC4A1*, and *EPB42* genes were found. According to ACMG standards (Richards et al., 2015), all the 10 novel mutations were classified as pathogenic, and have not been reported in any human gene mutation databases. The detailed evaluation is presented in Supplementary Table 2. Non-diagnosed HS may lead to complications, such as kernicterus, hemolytic anemia, and the development of gall stones. Thus, early diagnosis of HS is crucial to reduce the risk of complications later in life (Steward et al., 2014; Christensen et al., 2015). The 14 HS cases diagnosed in this study have an average age of 2.2 years (including 7 cases less than 1 year old), meaning that an earlier diagnosis is more conducive to reducing the risk of complications in these patients.

The analysis of all HS-associated mutations (including 144 from previous reports and 14 from this study) in Chinese patients showed that *ANK1* or *SPTB* was the major causative gene of HS cases, which is consistent with previous reports (Delaunay, 2007; Park et al., 2016; Wang et al., 2018; Qin et al., 2020), but only 5–10% of HS cases in Japan or Brazil were caused by *ANK1* mutations, implying that the geographical distribution of *ANK1* gene mutations is different. In addition, Chinese HS patients who carried *SLC4A1* mutations only account for 14%, which is close to the mutation incidence of Caucasian (15–20%) or Japanese patients (20%). Hughes et al. obtained a mice model by using N-ethyl-N-nitrosourea mutagenesis to generate random point mutations in exon 27 of Ank1 (E924X) in the mouse genome. They found that heterozygous mice have low RBC (MCV), reticulocytosis, and increased osmotic fragility, which are the clinical features of HS, while the homozygous mutant mice displayed severe hemolytic anemia, profound extramedullary hematopoiesis, stress erythropoiesis, and many pups died within the first 2 weeks of birth.

**FIGURE 3:**
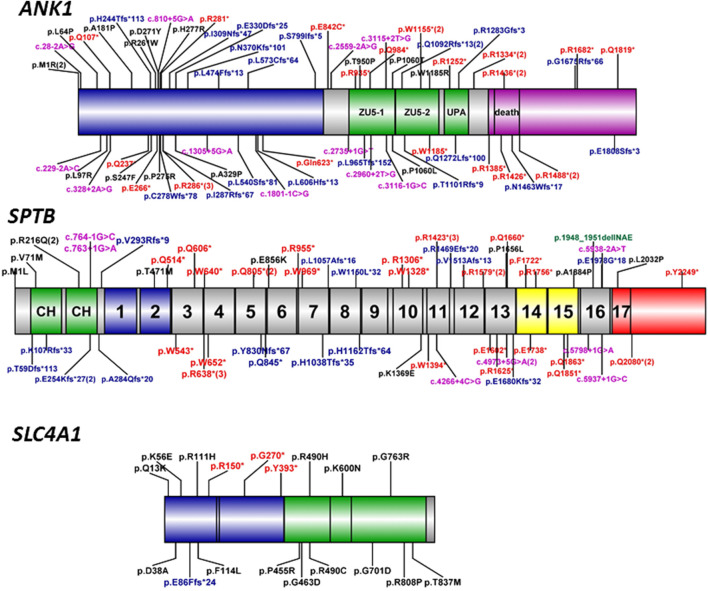
Schematic diagram of ankyrin, *ß*-spectrin, and band three protein domains with *ANK1, SPTB,* and *SLC4A1* mutations. Human erythroid ankyrin encoded by *ANK1* consists of an N-terminal membrane protein binding domain containing ankyrin repeats (blue box), a central spectrin-binding domain that involves two ZU5 and the UPA domains (green box), and a C-terminal regulatory domain that modulates the affinities of the other domains and contains a death domain (violet box). Human erythroid-spectrin protein encoded by *SPTB* mainly consists of two N-terminal actin-binding domains (green box) and seventeen spectrin repeats, of which repeats one and 2 (blue box) mediate α/β dimer formation, repeats 14 and 15 (yellow box) are the ankyrin-binding domain, and the last repeat is a tetramerization domain (red box). Human band three protein encoded by *SLC4A1* mainly consists of an N-terminal cytoplasmic domain (blue box) responsible for *ANK1* binding and the C-terminal domain (green box) spanning the lipid bilayer involved in anion transport. The different mutation types were labeled with different colors, black-missense, red-nonsense, blue-frameshift, violet-splicing.

Ankyrin-1 protein, encoded by *the ANK1* gene, is important for interacting with transmembrane proteins and the membrane skeleton of cells through *ß*-spectrin, band 3, and band 4.2 proteins. High affinity interaction is critical for the deformability and stability of the erythrocytes. Its deficiency leads to a decrease of spectrin assembly on the membrane, which causes anemia. *ANK1,* located on chromosome 8p11.2, contains 42 exons and encodes the ankyrin-1 protein (ANK1) with 1,881 amino acids (NM_020476.2), consisting of three main domains: an N-terminal membrane protein domain containing binding site for the band three membrane protein, a central domain binding the spectrin that involves two ZU5 and one UPA domain for interacting with the actin-spectrin cytoskeleton, and a regulatory domain of C-terminal, responsible for modulating the affinities of the other domains (Figure 3). In this study, five novel mutations were identified in *ANK1*, including two (p.Q107*, p. Q237*) on the N-terminal domain, three mutations on the central domain including one (p.Q984*) on ZU5-1, and two (p.Q1272Lfs*100, p. R1283Gfs*3) on UPA, which were all deleterious mutations, have been predicted to damage the binding with the band three membrane protein and the actin-sepctrin cytoskeleton resulting in the occurrence of HS.

To our knowledge, more than 190 *ANK1* mutations associated with HS have been described to date, including nonsense, missense, and splicing mutations, small or gross deletions, insertions, regulatory mutations, and complex rearrangements. In this study, we have identified 5 novel and two reported *ANK1* mutations including five nonsense and two frameshift mutations (Table 2). We then summarized and analyzed the mutational characteristics of *ANK1* mutations in Chinese HS patients and found that exon 8, 9, 26, and 28 of *ANK1* gene are high frequency mutant exons (Figure 2) and types of *ANK1* mutations in Chinese HS patients include nonsense mutations (36%), frameshift mutations (27%, containing small or gross deletions, insertions), missense mutations (18%), splicing abnormalities (15%) and start codon mutations (4%), but no regulatory mutations and complex rearrangements. All the *ANK1* mutations summarized in this study were heterozygous mutations, although autosomal recessive (AR) and non-dominant inheritance have been described in the HS patients with *ANK1* mutations by a few reports (Eber et al., 1996; Iolascon et al., 2019; Zamora and Schaefer, 2019). In addition, for the first time, we analyzed the distribution of *ANK1* mutations in the ankyrin-1 domain and found that the *ANK1* mutations in Chinese HS patients are mainly distributed in the N-terminal membrane protein binding domain, two ZU5 and the UPA domains, as well as the death domain. However, different types of *ANK1* mutations in different domains were presented as randomly distributed (Figure 3).

β-spectrin, one of the principal sepctrins of the assembly, is anchored to membrane proteins *via* ankyrin-1 by which spectrin plays a crucial role in the formation and stability of the erythrocyte membrane. The *SPTB* gene (MIM: 182,870), located on chromosome 14q23-q24.2, has a length of more than 100 kb and encodes *ß*-spectrin with 2,328 amino acids (NM_001024858), forming the cytoskeletal superstructure of the erythrocyte plasma membrane, which is comprised of two N-terminal calponin homology (CH) domains and seventeen spectrin repeats domains containing two domains mediating α/β dimer formation, two ankyrin-binding domains and a tetramerization domain (Figure 3) (Ipsaro et al., 2009). 185 *SPTB* mutations have been reported in the HGMD database. Unlike *ANK1* mutations, which were identified only in HS patients, *SPTB* mutations could result in other diseases including hereditary elliptocytosis and hereditary pyropoikilocytosis. Here, we have identified 5 novel and two reported *SPTB* mutations including four nonsense and four frameshift mutations only in the HS patients (Table 2). The five novel pathogenic mutations included two nonsense (p.W1328*, p. Q1660*) and three fromeshit (p.L1057Afs*16, p. W1150L*32, p. E1978G*18) located on the spectrin repeats domains of 7, 8, 10, 13, and 16, which resulted in truncation or premature termination of the protein causing loss of normal function thereby the occurrence of HS.

Some reports have pointed out that the *SPTB* mutations in HS patients are mainly distributed outside the tetramerization domain of the C-terminus in *ß*-spectrin (Park et al., 2016; Wang et al., 2018). Until now, 66 *SPTB* mutations (including the seven mutations identified in this study, see Supplementary Table 1) have been reported in 66 unrelated Chinese HS patients. Almost all these mutations were distributed outside the tetramerization domain except four mutations (c.6095T > C, c.6747C > G and two c.6238C > T, see Supplementary Table 1 and Figure 3), which might be associated with the HE phenotype, and only two mutations located on the spectrin repeats one and two domain. In addition, Most of these *SPTB* mutations are nonsense (32/66) and frameshift (20/73) mutations, which can be considered as null mutations and cause haploinsufficiency of *SPTB* and *ß*-spectrin deficiency in HS patients, and, interestingly, most of the nonsense mutations are mainly distributed in repeats 3–15 of erythroid-spectrin protein (Figure 3).

Band three or anion exchanger 1 (AE1) encoded by *SLC4A1* contains three distinct domains: an N-terminal band three cytoplasmic domain (residue 1–403) that is responsible for *ANK1* binding, a C-terminal domain (residues 404–882) that consists of 12–14 segments spanning the lipid bilayer involved in anion transport, and a short C-terminal cytoplasmic tail at the extreme (residues 883–911) with the function of binding to carbonic anhydrase II (Reithmeier et al., 2016). In this study, no *SLC4A1* mutation was found in our 14 cases. Until now, 176 *SLC4A1* mutations have been reported in the professional version of the HGMD database. Mariani et al. reported that band three deficiencies were the most common protein abnormalities (54%) in European HS patients (Mariani et al., 2008); however, only 18 mutations (11%) dispersed throughout the whole gene have been reported in Chinese HS patients (Supplementary Table 1 and Figure 3), and most of these *SLC4A1* mutations were missense mutations, this result is consistent with some previous reports (Yawata et al., 2000; Barneaud-Rocca et al., 2011; Reithmeier et al., 2016).

In addition, only one compound heterozygous mutation in the *SPTA1* gene and no *EPB42* mutations were reported in Chinese HS patients, indicating that pathogenic mutations of the two genes are relatively rare in the Chinese population.

This study found no significant differences in Hb level, reticulocytes, and total bilirubin among different groups of *ANK1*, *SPTB,* and *SLC4A1* genes. These results are consistent with a previous report (Qin et al., 2020). In addition, we also compared the clinical features among different types of mutations, but no significant differences were found among them either.

## Conclusion

This study has reported on 14 Chinese patients with suspected clinical features of HS and identified 14 pathogenic gene mutations (10 novel and four reported in *ANK1* and *SPTB*) by whole-exome sequencing. We clarified the mutational characteristics in Chinese patients with HS: *ANK1* gene mutation is the most major cause of HS followed by the *SPTB* gene. Findings also indicated that most mutations in *ANK1* or *SPTB* are heterozygous nonsense or frameshift mutations, which are also known as null mutations.

## Data Availability

The datasets presented in this study can be found in online repositories. The names of the repository/repositories and accession number(s) can be found below: GenBank (https://www.ncbi.nlm.nih.gov/Genbank) with accession number from MW812426 to MW812439.
