# Supplementary material for: Mutational Characteristics of Causative Genes in Chinese Hereditary Spherocytosis Patients: a Report on Fourteen Cases and a Review of the Literature
Source: Front Pharmacol. 2021 Jul 16;12:644352. doi: 10.3389/fphar.2021.644352 (PMC8322660; doi:10.3389/fphar.2021.644352)
Supplement: Supplementary file 1 [file Table1.DOCX]

**Supplementary table 1** Gene-mutations and clinical features in Chinese patients with hereditary spherocytosis

| **No** | **Gene** | **Exon** | **DNA change** | **Effect** | **Mutation type** | **Clinical features** | | | **Reference** |
| --- | --- | --- | --- | --- | --- | --- | --- | --- | --- |
|  |  |  |  |  |  | **Hb level (g/l)** | **Reticulocytes (%)** | **Total bilirubin (μmol/l)** |  |
| 1 | *ANK1* | 1^#^ | c.2T>G | p.M1R | Het. start codon mutation | – | – | – | (Peng et al., 2018) |
| 2 | *ANK1* | 1 | c.2T>G | p.M1R | Het. start codon mutation | – | – | – | (Wang et al., 2018) |
| 3 | *ANK1* | 1 | c.2T>G | p.M1R | Het. start codon mutation | 54 | 17.71 | 99.7 | (Qin et al., 2020) |
| 4 | *ANK1* | 2 | c.28-2A>G | oblig. splice site | Het. Splicing | 104 | – | 52.3 | (Qin et al., 2020) |
| 5 | *ANK1* | 3 | c.191T>C | p.L64P | Het. Missense | 74 | – | 177.2 | (Qin et al., 2020) |
| 6 | *ANK1* | 4 | c.229-2A>C | oblig. splice site | Het. Splicing | – | – | 118.1 | (Wang et al., 2017a) |
| 7 | *ANK1* | 4 | c.T290G | p.L97R | Het. Missense | 60 | 7.57 | 53.53 | (Hao et al., 2019) |
| **8** | ***ANK1*** | **4** | **c.319C>T** | **p.Q107*** | **Het. Nonsense** | **109** | **8.44** | **510.8** | **This study** |
| 9 | *ANK1* | 6 | c.328+2A>G | oblig. splice site | Het. Splicing | – | – | – | (Peng et al., 2018) |
| 10 | *ANK1* | 6 | c.541G > C | p.A181P | Het. Missense | – | – | – | (Peng et al., 2018) |
| **11** | ***ANK1*** | **7** | **c.709C>T** | **p.Q237*** | **Het. Nonsense** | **57** | **8.47** | **395** | **This study** |
| 12 | *ANK1* | 8 | c.725_728dupCACT | p.H244Tfs*113 | Het. Frameshift | – | – | – | (Wang et al., 2018) |
| 13 | *ANK1* | 8 | c.740C > T | p.S247F | Het. Missense | – | – | – | (Peng et al., 2018) |
| 14 | *ANK1* | 8 | c.781C > T | p.R261W | Het. Missense | – | – | – | (Peng et al., 2018) |
| 15 | *ANK1* | 8 | c.796G > T | p.E266* | Het. Nonsense | 60.2 | 8.7 | – | (Guan et al., 2018) |
| 16 | *ANK1* | 8 | c.810+5G>A | oblig. splice site | Het. Splicing | – | – | – | (Peng et al., 2018) |
| 17 | *ANK1* | 9 | c.811G > T | p.D271Y | Het. Missense | – | – | – | (Peng et al., 2018) |
| 18 | *ANK1* | 9 | c.824C>G | p.P275R | Het. Missense | 55 | 19 | – | (Xue et al., 2020) |
| 19 | *ANK1* | 9 | c.830A>G | p.H277R | Het. Missense | – | – | – | (Zhang and Xu, 2019) |
| 20 | *ANK1* | 9 | c.834_833insC | p.C278Wfs*78 | Het. Frameshift | 112 | 5.5 | 521.2 | (Jiang et al., 2016) |
| 21 | *ANK1* | 9 | c.841C>T | p.R281* | Het. Nonsense | 48 | 8.55 | 43.4 | (Hao et al., 2019) |
| 22 | *ANK1* | 9 | c.841C>T | p.R281* | Het. Nonsense | 80 | 9.32 | 82.1 | (Qin et al., 2020) |
| 23 | *ANK1* | 9 | c.856C>T | p.R286* | Het. Nonsense | – | – | – | (Wang et al., 2018) |
| 24 | *ANK1* | 9 | c.856C> T | p.R286* | Het. Nonsense | – | – | – | (Peng et al., 2018) |
| **25** | ***ANK1*** | **9** | **c.856C> T** | **p.R286*** | **Het. Nonsense** | **109** | **11.7** | **148.9** | **This study** |
| 26 | *ANK1* | 9 | c.858_862delAATCT | p.I287Rfs*67 | Het. Frameshift | 44 | 8.78 | 49.90 | (Gong et al., 2019) |
| 27 | *ANK1* | 9 | c.923dupC | p.I309Nfs*47 | Het. Frameshift | 108 | – | 57.8 | (Qin et al., 2020) |
| 28 | *ANK1* | 10 | c.985G>C | p.A329P | Het. Missense | – | – | – | (Zhang and Xu, 2019) |
| 29 | *ANK1* | 10 | c.990_991delGA | p.E330Dfs*25 | Het. Frameshift | – | – | – | (Peng et al., 2018) |
| 30 | *ANK1* | 11 | c.1109dupA | p.N370Kfs*101 | Het. Frameshift | – | – | – | (Wang et al., 2018) |
| 31 | *ANK1* | 12 | c.1305+5G>A | oblig. splice site | Het. Splicing | 73 | – | – | (Qin et al., 2020) |
| 32 | *ANK1* | 14 | c.1420delC | p.L474Ffs*13 | Het. Frameshift | 71 | 5.61 | – | (Qin et al., 2020) |
| 33 | *ANK1* | 15 | c.1616dupC | p.L540Sfs*81 | Het. Frameshift | 73 | – | – | (Qin et al., 2020) |
| 34 | *ANK1* | 16 | c.1717delC | p.L573Cfs*64 | Het. Frameshift | – | – | – | (Peng et al., 2018) |
| 35 | *ANK1* | 17 | c.1801-1C>G | oblig. splice site | Het. Splicing | 61 | 11.54 | 86.5 | (Sun et al., 2019) |
| 36 | *ANK1* | 17 | c.1814_1818  delCTTTG | p.L606Hfs*13 | Het. Frameshift | – | – | – | (Wang et al., 2018) |
| 37 | *ANK1* | 17 | c.1867C>T | p.Gln623* | Het. Nonsense | – | – | – | (Wang et al., 2018) |
| 38 | *ANK1* | 22 | c.2394_2397 del | p.S799Ifs*5 | Het. Frameshift | 148 | – | 53.4 | (Qin et al., 2020) |
| 39 | *ANK1* | 23 | c.2524G> T | p.E842C* | Het. Nonsense | – | – | – | (Peng et al., 2018) |
| 40 | *ANK1* | 24 | c.2559-2A>G | oblig. splice site | Het. Splicing | 73 | 11.05 | 78.8 | (Qin et al., 2020) |
| 41 | *ANK1* | 25 | c.2735+1G>T | oblig. splice site | Het. Splicing | – | – | – | (Wang et al., 2018) |
| 42 | *ANK1* | 26 | c.2803C>T | p.R935* | Het. Nonsense | – | – | – | (Wang et al., 2018) |
| 43 | *ANK1* | 26 | c.2848A> C | p.T950P | Het. Missense | – | – | – | (Peng et al., 2018) |
| 44 | *ANK1* | 26 | c.2891dupC | p.L965Tfs*152 | Het. Frameshift | 85 | 23.57 | 65.8 | (Qin et al., 2020) |
| **45** | ***ANK1*** | **26** | **c.2950C>T** | **p.Q984*** | **Het. Nonsense** | **64** | **12.2** | **38** | **This study** |
| 46 | *ANK1* | 26 | c.2960+2T>G | oblig. splice site | Het. Splicing | 67 | 3.09 | NA | (Luo et al., 2018) |
| 47 | *ANK1* | 27 | c.3115+2T>G | oblig. splice site | Het. Splicing | – | – | – | (Wang et al., 2018) |
| 48 | *ANK1* | 28 | c.3116-1G>C | oblig. splice site | Het. Splicing | – | – | – | (Peng et al., 2018) |
| 49 | *ANK1* | 28 | c.3178C> A | p.P1060T | Het. Missense | – | – | – | (Peng et al., 2018) |
| 50 | *ANK1* | 28 | c.3179C>T | p.P1060L | Het. Missense | 89 | 17.18 | 93.7 | (Qin et al., 2020) |
| 51 | *ANK1* | 28 | c.3275delA | p.Q1092Rfs*13 | Het. Frameshift | – | – | – | (Wang et al., 2018) |
| 52 | *ANK1* | 28 | c.3275delA | p.Q1092Rfs*13 | Het. Frameshift | 62 | 10.90 | 39.60 | (Gong et al., 2019) |
| 53 | *ANK1* | 28 | c.3302_3305delinsGAGTGCCGGAGAATGCCG | p.T1101Rfs*9 | Het. Frameshift | – | – | – | (Wang et al., 2018) |
| 54 | *ANK1* | 29 | c.3464G>A | p.W1155* | Het. Nonsense | – | – | – | (Wang et al., 2018) |
| 55 | *ANK1* | 29 | c.3464G>A | p.W1155* | Het. Nonsense | – | – | – | (Peng et al., 2018) |
| 56 | *ANK1* | 30 | c.3553T> C | p.W1185R | Het. Missense | – | – | – | (Peng et al., 2018) |
| 57 | *ANK1* | 30 | c.3554G>A | p.W1185* | Het. Nonsense | – | – | – | (Wang et al., 2018) |
| **58** | ***ANK1*** | **31** | **c.3754C>T** | **p.R1252*** | **Het. Nonsense** | **50** | **4.1** | **22** | **This study** |
| **59** | ***ANK1*** | **31** | **c.3813_3823del** | **p.Q1272Lfs*100** | **Het. Frameshift** | **54** | **6.3** | **179** | **This study** |
| **60** | ***ANK1*** | **31** | **c.3847delA** | **p.R1283Gfs*3** | **Het. Frameshift** | **76** | **17.02** | **55.7** | **This study** |
| 61 | *ANK1* | 33 | c.4000C>T | p.R1334* | Het. Nonsense | – | – | – | (Wang et al., 2018) |
| 62 | *ANK1* | 33 | c.4000C>T | p.R1334* | Het. Nonsense | 79 | 17.88 | 47.5 | (Qin et al., 2020) |
| 63 | *ANK1* | 34 | c.4153C>T | p.R1385* | Het. Nonsense | – | – | – | (Wang et al., 2018) |
| 64 | *ANK1* | 34 | c.4276C>T | p.R1426* | Het. Nonsense | 58 | – | – | (Wang et al., 2017b) |
| 65 | *ANK1* | 36 | c.4306C>T | p.R1436* | Het. Nonsense | 65 | 9.32 | 100.00 | (Gong et al., 2019) |
| 66 | *ANK1* | 36 | c.4306C>T | p.R1436* | Het. Nonsense | – | – | – | (Wang et al., 2018) |
| 67 | *ANK1* | 36 | c.4387_4390  delAACA | p.N1463Wfs*17 | Het. Frameshift | – | – | – | (Qin et al., 2020) |
| 68 | *ANK1* | 37 | c.4462C>T | p.R1488* | Het. Nonsense | 26 | – | – | (Qin et al., 2020) |
| 69 | *ANK1* | 37 | c.4462C>T | p.R1488* | Het. Nonsense | 72 | – | – | (Qin et al., 2020) |
| 70 | *ANK1* | 38 | c.5022dupA | p.G1675Rfs*66 | Het. Frameshift | – | – | – | (Peng et al., 2018) |
| 71 | *ANK1* | 38 | c.5044C>T | p.R1682* | Het. Nonsense | – | – | – | (Peng et al., 2018) |
| 72 | *ANK1* | 40 | c.5422delG | p.E1808Sfs*3 | Het. Frameshift | 40 | 8.2 | 103.7 | (Li et al., 2019a) |
| 73 | *ANK1* | 40 | c.5455C>T | p.Q1819* | Het. Nonsense | – | – | – | (Wang et al., 2018) |
| 74 | *SPTB* | 1 | c.1A>C | start codon defect | Het. start codon mutation | – | – | – | (Wang et al., 2018) |
| 75 | *SPTB* | 2 | c.173dupA | p.T59Dfs*113 | Het. Frameshift | – | – | – | (Wang et al., 2018) |
| 76 | *SPTB* | 2 | c.211G>A | p.V71M | Het. Missense | 85 | 1.1 | – | (Xue et al., 2020) |
| 77 | *SPTB* | 3 | c.318delG | p.K107Rfs*33 | Het. Frameshift | 50 | 1.93 | 53.9 | (Gong et al., 2019) |
| 78 | *SPTB* | 5 | c.647G>A | p.R216Q | Het. Missense | – | – | – | (Wang et al., 2018) |
| 79 | *SPTB* | 5 | c.647G>A | p.R216Q | Het. Missense | 81 | 12.34 | – | (Qin et al., 2020) |
| 80 | *SPTB* | 6 | c.759delC | p.E254Kfs*27 | Het. Frameshift | – | – | – | (Peng et al., 2018) |
| 81 | *SPTB* | 6 | c.759delC | p.E254Kfs*27 | Het. Frameshift | – | – | – | (Wang et al., 2018) |
| 82 | *SPTB* | 6 | c.763+1G>A | oblig. splice site | Het. Splicing | – | – | – | (Wang et al., 2018) |
| 83 | *SPTB* | 7 | c.764-1G>C | oblig. splice site | Het. Splicing | 74 | 13.91 | 109 | (Maidina et al., 2019) |
| 84 | *SPTB* | 7 | c.850delG | p.A284Qfs*20 | Het. Frameshift | – | – | – | (Peng et al., 2018) |
| 85 | *SPTB* | 8 | c.877_887delinsAGAC | p.V293Rfs*9 | Het. Frameshift | – | – | – | (Wang et al., 2018) |
| 86 | *SPTB* | 11 | c.1412C>T | p.T471M | Het. Missense | 81 | 15.27 | 25.1 | (Qin et al., 2020) |
| 87 | *SPTB* | 11 | c.1540C>T | p.Q514* | Het. Nonsense | – | – | – | (Peng et al., 2018) |
| 88 | *SPTB* | 11 | c.1628G>A | p.W543* | Het. Nonsense | – | – | – | (Peng et al., 2018) |
| 89 | *SPTB* | 13 | c.1816C>T | p.Q606* | Het. Nonsense | 93 | – | 138.7 | (Qin et al., 2020) |
| 90 | *SPTB* | 13 | c.1912C>T | p.R638* | Het. Nonsense | – | – | – | (Peng et al., 2018) |
| 91 | *SPTB* | 13 | c.1912C>T | p.R638* | Het. Nonsense | 10.8 | 17.44 | 76.7 | (Qin et al., 2020) |
| 92 | *SPTB* | 13 | c.1912C>T | p.R638* | Het. Nonsense | 59 | 7.63 | 202 | This study |
| 93 | *SPTB* | 13 | c.1920G>A | p.W640* | Het. Nonsense | – | – | – | (Wang et al., 2018) |
| 94 | *SPTB* | 13 | c.1955G>A | p.W652* | Het. Nonsense | – | – | – | (Wang et al., 2018) |
| 95 | *SPTB* | 13 | c.2413 C > T | p.Q805* | Het. Nonsense | 85 | 7.89 | 111.8 | (Li et al., 2019b) |
| 96 | *SPTB* | 13 | c.2413C>T | p.Q805* | Het. Nonsense | – | – | – | (Peng et al., 2018) |
| 97 | *SPTB* | 13 | c.2488_2491delTACC | p.Y830Nfs*67 | Het. Frameshift | – | – | – | (Peng et al., 2018) |
| 98 | *SPTB* | 13 | c.2533C>T | p.Q845* | Het. Nonsense | 64 | – | – | (Qin et al., 2020) |
| 99 | *SPTB* | 13 | c.2566G>A | p.E856K | Het. Missense | – | – | – | (Peng et al., 2018) |
| 100 | *SPTB* | 15 | c.2863C>T | p.R955* | Het. Nonsense | – | – | – | (Wang et al., 2018) |
| 101 | *SPTB* | 15 | c.2907G>A | p.W969* | Het. Nonsense | – | – | – | (Peng et al., 2018) |
| 102 | *SPTB* | 15 | c.3111dupA | p.H1038Tfs*35 | Het. Frameshift | – | – | – | (Wang et al., 2018) |
| 103 | *SPTB* | 15 | c.3168dupG | p.L1057Afs*16 | Het. Frameshift | 56 | 5.78 | 272.3 | This study |
| 104 | *SPTB* | 15 | c.3448dupT | p.W1150L*32 | Het. Frameshift | 54 | 8.88 | 146.4 | This study |
| 105 | *SPTB* | 15 | c.3484delC | p.H1162Tfs*64 | Het. Frameshift | 86 | 6.35 | 103 | (Gong et al., 2019) |
| 106 | *SPTB* | 18 | c.3916C>T | p. R1306* | Het. Nonsense | – | – | – | (Wang et al., 2018) |
| 107 | *SPTB* | 18 | c.3984G>A | p.W1328* | Het. Nonsense | 54 | 14.4 | 34.1 | This study |
| 108 | *SPTB* | 19 | c.4105A>G | p.K1369E | Het. Missense | 91 | 11.78 | 56.1 | (Qin et al., 2020) |
| 109 | *SPTB* | 19 | c.4181G>A | p.W1394* | Het. Nonsense | 90 | 8.9 | – | (Xue et al., 2020) |
| 110 | *SPTB* | 19 | c.4266+4C>G | oblig. splice site | Het. Splicing | – | – | – | (Peng et al., 2018) |
| 111 | *SPTB* | 20 | c.4267C>T | p.R1423* | Het. Nonsense | – | – | – | (Peng et al., 2018) |
| 112 | *SPTB* | 20 | c.4267C>T | p. R1423* | Het. Nonsense | – | – | – | (Wang et al., 2018) |
| 113 | *SPTB* | 20 | c.4267C>T | p. R1423* | Het. Nonsense | – | – | – | (Qin et al., 2020) |
| 114 | *SPTB* | 20 | c.4403_4404 insGA | p.R1469Efs*20 | Het. Frameshift | 87 | 12.86 | 62.2 | (Qin et al., 2020) |
| 115 | *SPTB* | 21 | c.4538_4539 delTG | p.V1513Afs*13 | Het. Frameshift | 68 | 14.45 | 108.9 | (Qin et al., 2020) |
| 116 | *SPTB* | 22 | c.4735C>T | p.R1579* | Het. Nonsense | – | – | – | (Wang et al., 2018) |
| 117 | *SPTB* | 22 | c.4735C>T | p.R1579* | Het. Nonsense | 87 | – | 80.7 | (Qin et al., 2020) |
| 118 | *SPTB* | 22 | c.4804G>T | p.E1602* | Het. Nonsense | 117 | 7.88 | 133.9 | (Qin et al., 2020) |
| 119 | *SPTB* | 23 | c.C4873T | p.R1625* | Het. Nonsense | 125 | – | 73.1 | (Shen et al., 2019) |
| 120 | *SPTB* | 23 | c.4967C>T | p.P1656L | Het. Missense | – | – | – | (Peng et al., 2018) |
| 121 | *SPTB* | 23 | c.4973+5G>A | oblig. splice site | Het. Splicing | – | – | – | (Wang et al., 2018) |
| 122 | *SPTB* | 23 | c.4973+5G>A | oblig. splice site | Het. Splicing | 68 | 8.6 | – | (Xue et al., 2020) |
| 123 | *SPTB* | 24 | c.4978C>T | p.Q1660* | Het. Nonsense | 92 | 12.92 | 54.4 | This study |
| 124 | *SPTB* | 24 | c.5038delG | p.E1680Kfs*32 | Het. Frameshift | – | – | – | (Qin et al., 2020) |
| 125 | *SPTB* | 24 | c.5165_c.5166delTT | p.F1722* | Het. Nonsense | 64 | 9.7 | 66.5 | (Zhang et al., 2020) |
| 126 | *SPTB* | 25 | c.5212G>T | p.E1738* | Het. Nonsense | 109 | 16.83 | 74.9 | (Qin et al., 2020) |
| 127 | *SPTB* | 25 | c.5266C>T | p.R1756* | Het. Nonsense | 60 | 3.56 | 484.7 | This study |
| 128 | *SPTB* | 25 | c.5551C>T | p.Q1851* | Het. Nonsense | 91 | – | – | (Qin et al., 2020) |
| 129 | *SPTB* | 26 | c.5587C>T | p.Q1863* | Het. Nonsense | – | – | – | (Peng et al., 2018) |
| 130 | *SPTB* | 26 | c.5650G > C | p.A1884P | Het. Missense | 88 | 4.00 | 51.60 | (Fan et al., 2019) |
| 131 | *SPTB* | 26 | c.5798+1G>A | oblig. splice site | Het. Splicing | 60 | – | 114.7 | (Liu et al., 2020) |
| 132 | *SPTB* | 27 | c.5844_5855delCAATGCAGAGAT | p.1948_1951delINAE | Het. Deletion | – | – | – | (Peng et al., 2018) |
| 133 | *SPTB* | 27 | c.5933_5934delAG | p.E1978G*18 | Het. Frameshift | 79 | 14.23 | 87.7 | This study |
| 134 | *SPTB* | 27 | c.5937+1G>C | oblig. splice site | Het. Splicing | 56 | 13.18 | 117.9 | (Qin et al., 2020) |
| 135 | *SPTB* | 28 | c.5938-2A>T | oblig. splice site | Het. Splicing | 99 | 7.4 | 70.9 | (Qin et al., 2020) |
| 136 | *SPTB* | 29 | c.6095T>C | p.L2032P | Hom. Missense | 55 | 6 | – | (Yang et al., 2019) |
| 137 | *SPTB* | 30 | c.6238C>T | p.Q2080* | Het. Nonsense | – | – | – | (Peng et al., 2018) |
| 138 | *SPTB* | 30 | c.6238C>T | p.Q2080* | Het. Nonsense | – | – | – | (Wang et al., 2018) |
| 139 | *SPTB* | 33 | c.6747C>G | p.Y2249* | Het. Nonsense | – | – | – | (Wang et al., 2018) |
| 140 | *SLC4A1* | 3 | c.37G>A | p.Q13K | Het. Missense | – | – | – | (Zhang and Xu, 2019) |
| 141 | *SLC4A1* | 4 | c.113A>C | p.D38A | Het. Missense | – | – | – | (Ma et al., 2018b) |
| 142 | *SLC4A1* | 4 | c.166A>G | p.K56E | Het. Missense | – | – | – | (Ma et al., 2018b) |
| 143 | *SLC4A1* | 5 | c.256-258delinsTTCTC | p.E86Ffs*24 | Het. Frameshift | 94 | 5.6 | – | (Xue et al., 2020) |
| 144 | *SLC4A1* | 5 | c.332G>A | p.R111H | Het. Missense | 56 | 20.3 | 45.2 | (Qin et al., 2020) |
| 145 | *SLC4A1* | 5 | c.340T>C | p.F114L | Het. Missense | – | – | – | (Zhang and Xu, 2019) |
| 146 | *SLC4A1* | 6 | c.448C>T | p.R150* | Het. Nonsense | – | – | 257 | (Qin et al., 2020) |
| 147 | *SLC4A1* | 9 | c.808G>T | p.G270* | Het. Nonsense | – | – | – | (Peng et al., 2018) |
| 148 | *SLC4A1* | 11 | c.1179C> A | p.Y393* | Het. Nonsense | – | – | – | (Peng et al., 2018) |
| 149 | *SLC4A1* | 12 | c.1363G>A | p.P455R | Het. Missense | 120 | – | – | (Qin et al., 2020) |
| 150 | *SLC4A1* | 12 | c.1388G>A | p.G463D | Het. Missense | – | – | – | (Wang et al., 2018) |
| 151 | *SLC4A1* | 13 | c.1468C>T | p.R490C | Het. Missense | – | – | – | (Wang et al., 2018) |
| 152 | *SLC4A1* | 13 | c.1469G > A | p.R490H | Het. Missense | 114 | – | 74 | (Shen et al., 2019) |
| 153 | *SLC4A1* | 14 | c.1800G>T | p.K600N | Het. Missense | – | – | – | (Wang et al., 2018) |
| 154 | *SLC4A1* | 17 | c.2102G>A | p.G701D | Het. Missense | – | – | – | (Peng et al., 2018) |
| 155 | *SLC4A1* | 17 | c.2287G>A | p.G763R | Het. Missense | – | – | – | (Peng et al., 2018) |
| 156 | *SLC4A1* | 18 | c.2423G>C | p.R808P | Het. Missense | – | – | – | (Wang et al., 2018) |
| 157 | *SLC4A1* | 19 | c.2510C>T | p.T837M | Het. Missense | – | – | – | (Peng et al., 2018) |
| 158 | *SPTA1* | 40/  46 | c.5572C>G/6531-12C>T | LELY | α^LELY^ | 48.9 | 7 | 178.1 | (Ma et al., 2018a) |
|  |  | 2 | c.161A>C | p.H54P | Het. Missense |  |  |  |  |

Hom.: Homozygous; Het.: Heterozygous; Hb: hemoglobin;

The variants shown in this Table are described using the NM_020476.3, NM_001024858.3, NM_000342.3 and NM_003126.2 for ANK1, SPTB, SLC4A1 and SPTA1 transcript reference sequence, respectively.

^#^This mutation is described using the NM_001142446.

**References**

Fan, L.L., Liu, J.S., Huang, H., Du, R., and Xiang, R. (2019). Whole exome sequencing identified a novel mutation (p.Ala1884Pro) of beta-spectrin in a Chinese family with hereditary spherocytosis. *J Gene Med* 21(2-3)**,** e3073. doi: 10.1002/jgm.3073.

Gong, J., He, X.L., Zou, R.Y., Chen, K.K., You, Y.L., Zou, H., et al. (2019). [Clinical characteristics and genetic analysis of hereditary spherocytosis caused by mutations of ANK1 and SPTB genes]. *Zhongguo Dang Dai Er Ke Za Zhi* 21(4)**,** 370-374.

Guan, H., Liang, X., Zhang, R., Wang, H., Liu, W., Yang, J., et al. (2018). Identification of a de novo ANK1 mutation in a Chinese family with hereditary spherocytosis. *Hematology* 23(6)**,** 357-361. doi: 10.1080/10245332.2017.1398210.

Hao, L., Li, S., Ma, D., Chen, S., Zhang, B., Xiao, D., et al. (2019). Two novel ANK1 loss-of-function mutations in Chinese families with hereditary spherocytosis. *J Cell Mol Med* 23(6)**,** 4454-4463. doi: 10.1111/jcmm.14343.

Jiang, M., Lu, J., Zhong, Y., Wang, Y., and Yang, C. (2016). [Identification of a novel ANK1 gene mutation in a newborn with hereditary spherocytosis]. *Zhonghua Yi Xue Yi Chuan Xue Za Zhi* 33(1)**,** 44-47. doi: 10.3760/cma.j.issn.1003-9406.2016.01.011.

Li, D., Li, B., Li, S., Li, W., Wang, Y., and Guo, X. (2019a). [Analysis of ANK1 gene mutation in a family with hereditary spherocytosis type ]. *Zhonghua Yi Xue Yi Chuan Xue Za Zhi* 36(10)**,** 999-1001. doi: 10.3760/cma.j.issn.1003-9406.2019.10.012.

Li, Y., Yang, Y., Yang, W.R., Li, J.P., Peng, G.X., Song, L., et al. (2019b). Next generation sequencing reveals co-existence of hereditary spherocytosis and Dubin-Johnson syndrome in a Chinese gril: A case report. *World J Clin Cases* 7(20)**,** 3303-3309. doi: 10.12998/wjcc.v7.i20.3303.

Liu, H., Huang, J., Jiang, Y., Guo, L., Xiao, H., and Wang, H. (2020). [Hereditary spherocytosis due to a novel c.5798+1G>A variant of the SPTB gene]. *Zhonghua Yi Xue Yi Chuan Xue Za Zhi* 37(1)**,** 17-20. doi: 10.3760/cma.j.issn.1003-9406.2020.01.005.

Luo, Y., Li, Z., Huang, L., Tian, J., Xiong, M., and Yang, Z. (2018). Spectrum of Ankyrin Mutations in Hereditary Spherocytosis: A Case Report and Review of the Literature. *Acta Haematol* 140(2)**,** 77-86. doi: 10.1159/000492024.

Ma, S., Deng, X., Liao, L., Deng, Z., Qiu, Y., Wei, H., et al. (2018a). Analysis of the causes of the misdiagnosis of hereditary spherocytosis. *Oncol Rep* 40(3)**,** 1451-1458. doi: 10.3892/or.2018.6578.

Ma, S.Y., Liao, L., He, B.J., and Lin, F.Q. (2018b). [Application of High Resolution Melting Curve Analysis in Detection of SLC4A1 Gene Mutation in Patients with Hereditary Spherocytosis]. *Zhongguo Shi Yan Xue Ye Xue Za Zhi* 26(6)**,** 1826-1830. doi: 10.7534/j.issn.1009-2137.2018.06.041.

Maidina, E., Wang, Y.C., Mao, M., and Li, Y. (2019). [A case report of SPTB new mutation in hereditary spherocytosis and literature review]. *Zhonghua Xue Ye Xue Za Zhi* 40(2)**,** 155. doi: 10.3760/cma.j.issn.0253-2727.2019.02.013.

Peng, G.X., Yang, W.R., Zhao, X., Jin, L.P., Zhang, L., Zhou, K., et al. (2018). [The characteristic of hereditary spherocytosis related gene mutation in 37 Chinese hereditary spherocytisis patients]. *Zhonghua Xue Ye Xue Za Zhi* 39(11)**,** 898-903. doi: 10.3760/cma.j.issn.0253-2727.2018.11.005.

Qin, L., Nie, Y., Zhang, H., Chen, L., Zhang, D., Lin, Y., et al. (2020). Identification of new mutations in patients with hereditary spherocytosis by next-generation sequencing. *J Hum Genet* 65(4)**,** 427-434. doi: 10.1038/s10038-020-0724-z.

Shen, H., Huang, H., Luo, K., Yi, Y., and Shi, X. (2019). Two different pathogenic gene mutations coexisted in the same hereditary spherocytosis family manifested with heterogeneous phenotypes. *BMC Med Genet* 20(1)**,** 90. doi: 10.1186/s12881-019-0826-7.

Sun, Q., Xie, Y., Wu, P., Li, S., Hua, Y., Lu, X., et al. (2019). Targeted next-generation sequencing identified a novel ANK1 mutation associated with hereditary spherocytosis in a Chinese family. *Hematology* 24(1)**,** 583-587. doi: 10.1080/16078454.2019.1650873.

Wang, R., Yang, S., Xu, M., Huang, J., Liu, H., Gu, W., et al. (2018). Exome sequencing confirms molecular diagnoses in 38 Chinese families with hereditary spherocytosis. *Sci China Life Sci* 61(8)**,** 947-953. doi: 10.1007/s11427-017-9232-6.

Wang, X., Mao, L., Shen, N., Peng, J., Zhu, Y., Hu, Q., et al. (2017a). An ANK1 IVS3-2A>C mutation causes exon 4 skipping in two patients from a Chinese family with hereditary spherocytosis. *Oncotarget* 8(68)**,** 113282-113286. doi: 10.18632/oncotarget.22936.

Wang, X., Yi, B., Mu, K., Shen, N., Zhu, Y., Hu, Q., et al. (2017b). Identification of a novel de novo ANK1 R1426* nonsense mutation in a Chinese family with hereditary spherocytosis by NGS. *Oncotarget* 8(57)**,** 96791-96797. doi: 10.18632/oncotarget.18243.

Xue, J., He, Q., Xie, X.J., Su, A.L., and Cao, S.B. (2020). A clinical and experimental study of adult hereditary spherocytosis in the Chinese population. *Kaohsiung J Med Sci* 36(7)**,** 552-560. doi: 10.1002/kjm2.12198.

Yang, K., Ren, Q., Wu, Y., Zhou, Y., and Yin, X. (2019). A Case of Hereditary Spherocytosis Caused by a Novel Homozygous Mutation in the SPTB Gene Misdiagnosed as beta-Thalassemia Intermedia Due to a KLF1 Gene Mutation. *Hemoglobin* 43(2)**,** 140-144. doi: 10.1080/03630269.2019.1620764.

Zhang, Y.G., and Xu, Z.L. (2019). [Clinical and genetic features of children with hereditary spherocytosis: an analysis of 4 cases]. *Zhongguo Dang Dai Er Ke Za Zhi* 21(1)**,** 29-32.

Zhang, Y.M., Zhang, X.R., Shao, S.M., Liu, J., Zeng, C.M., and Han, Y. (2020). [A case of neonatal hereditary spherocytosis characterized by hydrops fetalis]. *Zhonghua Er Ke Za Zhi* 58(5)**,** 418-420. doi: 10.3760/cma.j.cn112140-20200113-00030.
