# Supplementary material for: Mutational Characteristics of Causative Genes in Chinese Hereditary Spherocytosis Patients: a Report on Fourteen Cases and a Review of the Literature
Source: Front Pharmacol. 2021 Jul 16;12:644352. doi: 10.3389/fphar.2021.644352 (PMC8322660; doi:10.3389/fphar.2021.644352)
Supplement: Supplementary file 2 [file Table2.DOCX]

**Supplementary table 2** Pathogenicity assessment of the 10 novel variants according to ACMG guidelines.

| Patient number | Variant | ACMG criteria evaluation | |
| --- | --- | --- | --- |
| P4 | *SPTB* c.3168dupG | Very strong criterion | PVS1-null variant (frameshift) in *SPTB* gene where LOF is a known mechanism of HS. |
|  |  | Moderate criterion | PM2-absent from controls in Exome Sequencing Project, 1000 Genomes and Exome Aggregation Consortium.  PM4-protein length changes as a result of in-frame deletions/insertions in a nonrepeat region or stop-loss variants |
|  |  | Supportive criterion | PP3-multiple lines of computational evidence support a deleterious effect on the gene or gene product.  PP4-patient’s phenotype or family history is highly specific for a disease with a single genetic etiology |
| P5 | *SPTB* c.4978C>T | Very strong criterion | PVS1-null variant (nonsense) in *SPTB* gene where LOF is a known mechanism of HS. |
|  |  | Strong criterion | PS2-de novo variant (both maternity and paternity confirmed) and no family history. |
|  |  | Moderate criterion | PM2-absent from controls in Exome Sequencing Project, 1000 Genomes and Exome Aggregation Consortium.  PM4-protein length changes as a result of in-frame deletions/insertions in a nonrepeat region or stop-loss variants |
|  |  | Supportive criterion | PP3-multiple lines of computational evidence support a deleterious effect on the gene or gene product.  PP4-patient’s phenotype or family history is highly specific for a disease with a single genetic etiology. |
| P6 | *ANK1* c.319C>T | Very strong criterion | PVS1-null variant (nonsense) in *ANK1* gene where LOF is a known mechanism of HS. |
|  |  | Strong criterion | PS2-de novo variant (both maternity and paternity confirmed) and no family history. |
|  |  | Moderate criterion | PM2-absent from controls in Exome Sequencing Project, 1000 Genomes and Exome Aggregation Consortium.  PM4-protein length changes as a result of in-frame deletions/insertions in a nonrepeat region or stop-loss variants |
|  |  | Supportive criterion | PP3-multiple lines of computational evidence support a deleterious effect on the gene or gene product.  PP4-patient’s phenotype or family history is highly specific for a disease with a single genetic etiology. |
| P7 | *SPTB* c.5933_5934delAG | Very strong criterion | PVS1-null variant (frameshift) in *SPTB* gene where LOF is a known mechanism of HS. |
|  |  | Strong criterion | PS2-de novo variant (both maternity and paternity confirmed) and no family history. |
|  |  | Moderate criterion | PM2-absent from controls in Exome Sequencing Project, 1000 Genomes and Exome Aggregation Consortium.  PM4-protein length changes as a result of in-frame deletions/insertions in a nonrepeat region or stop-loss variants |
|  |  | Supportive criterion | PP3-multiple lines of computational evidence support a deleterious effect on the gene or gene product.  PP4-patient’s phenotype or family history is highly specific for a disease with a single genetic etiology. |
| P8 | *ANK1* c.709C>T | Very strong criterion | PVS1-null variant (nonsense) in *ANK1* gene where LOF is a known mechanism of HS. |
|  |  | Moderate criterion | PM2-absent from controls in Exome Sequencing Project, 1000 Genomes and Exome Aggregation Consortium.  PM4-protein length changes as a result of in-frame deletions/insertions in a nonrepeat region or stop-loss variants |
|  |  | Supportive criterion | PP1- Cosegregation with HS in multiple affected family members in *ANK1* gene.  PP3-multiple lines of computational evidence support a deleterious effect on the gene or gene product.  PP4-patient’s phenotype or family history is highly specific for a disease with a single genetic etiology. |
| P9 | *ANK1* c.2950C>T | Very strong criterion | PVS1-null variant (nonsense) in *ANK1* gene where LOF is a known mechanism of HS. |
|  |  | Strong criterion | PS2-de novo variant (both maternity and paternity confirmed) and no family history. |
|  |  | Moderate criterion | PM2-absent from controls in Exome Sequencing Project, 1000 Genomes and Exome Aggregation Consortium.  PM4-protein length changes as a result of in-frame deletions/insertions in a nonrepeat region or stop-loss variants |
|  |  | Supportive criterion | PP3-multiple lines of computational evidence support a deleterious effect on the gene or gene product.  PP4-patient’s phenotype or family history is highly specific for a disease with a single genetic etiology. |
| P10 | *ANK1* c.3813_3823del | Very strong criterion | PVS1-null variant (frameshift) in *ANK1* gene where LOF is a known mechanism of HS. |
|  |  | Strong criterion | PS2-de novo variant (both maternity and paternity confirmed) and no family history. |
|  |  | Moderate criterion | PM2-absent from controls in Exome Sequencing Project, 1000 Genomes and Exome Aggregation Consortium.  PM4-protein length changes as a result of in-frame deletions/insertions in a nonrepeat region or stop-loss variants |
|  |  | Supportive criterion | PP3-multiple lines of computational evidence support a deleterious effect on the gene or gene product.  PP4-patient’s phenotype or family history is highly specific for a disease with a single genetic etiology. |
| P11 | *SPTB* c.3984G>A | Very strong criterion | PVS1-null variant (nonsense) in *SPTB* gene where LOF is a known mechanism of HS. |
|  |  | Moderate criterion | PM2-absent from controls in Exome Sequencing Project, 1000 Genomes and Exome Aggregation Consortium.  PM4-protein length changes as a result of in-frame deletions/insertions in a nonrepeat region or stop-loss variants |
|  |  | Supportive criterion | PP3-multiple lines of computational evidence support a deleterious effect on the gene or gene product.  PP4-patient’s phenotype or family history is highly specific for a disease with a single genetic etiology |
| P12 | *SPTB* c.3448dupT | Very strong criterion | PVS1-null variant (frameshift) in *SPTB* gene where LOF is a known mechanism of HS. |
|  |  | Moderate criterion | PM2-absent from controls in Exome Sequencing Project, 1000 Genomes and Exome Aggregation Consortium.  PM4-protein length changes as a result of in-frame deletions/insertions in a nonrepeat region or stop-loss variants |
|  |  | Supportive criterion | PP3-multiple lines of computational evidence support a deleterious effect on the gene or gene product.  PP4-patient’s phenotype or family history is highly specific for a disease with a single genetic etiology |
| P14 | *ANK1* c.3847delA | Very strong criterion | PVS1-null variant (frameshift) in *ANK1* gene where LOF is a known mechanism of HS. |
|  |  | Strong criterion | PS2-de novo variant (both maternity and paternity confirmed) and no family history. |
|  |  | Moderate criterion | PM2-absent from controls in Exome Sequencing Project, 1000 Genomes and Exome Aggregation Consortium.  PM4-protein length changes as a result of in-frame deletions/insertions in a nonrepeat region or stop-loss variants |
|  |  | Supportive criterion | PP3-multiple lines of computational evidence support a deleterious effect on the gene or gene product.  PP4-patient’s phenotype or family history is highly specific for a disease with a single genetic etiology. |

LOF: Loss of function; HS: hereditary spherocytosis;

The variants shown in Supplementary table 2 are described using the NM_020476.2 and NM_001024858.2 for *ANK1* and *SPTB* transcript reference sequence, respectively.
