# Supplementary material for: Mutational Characteristics of Causative Genes in Chinese Hereditary Spherocytosis Patients: a Report on Fourteen Cases and a Review of the Literature
Source: Front Pharmacol. 2021 Jul 16;12:644352. doi: 10.3389/fphar.2021.644352 (PMC8322660; doi:10.3389/fphar.2021.644352)
Supplement: Supplementary file 3 [file DataSheet1.docx]

**Supplementary figures**


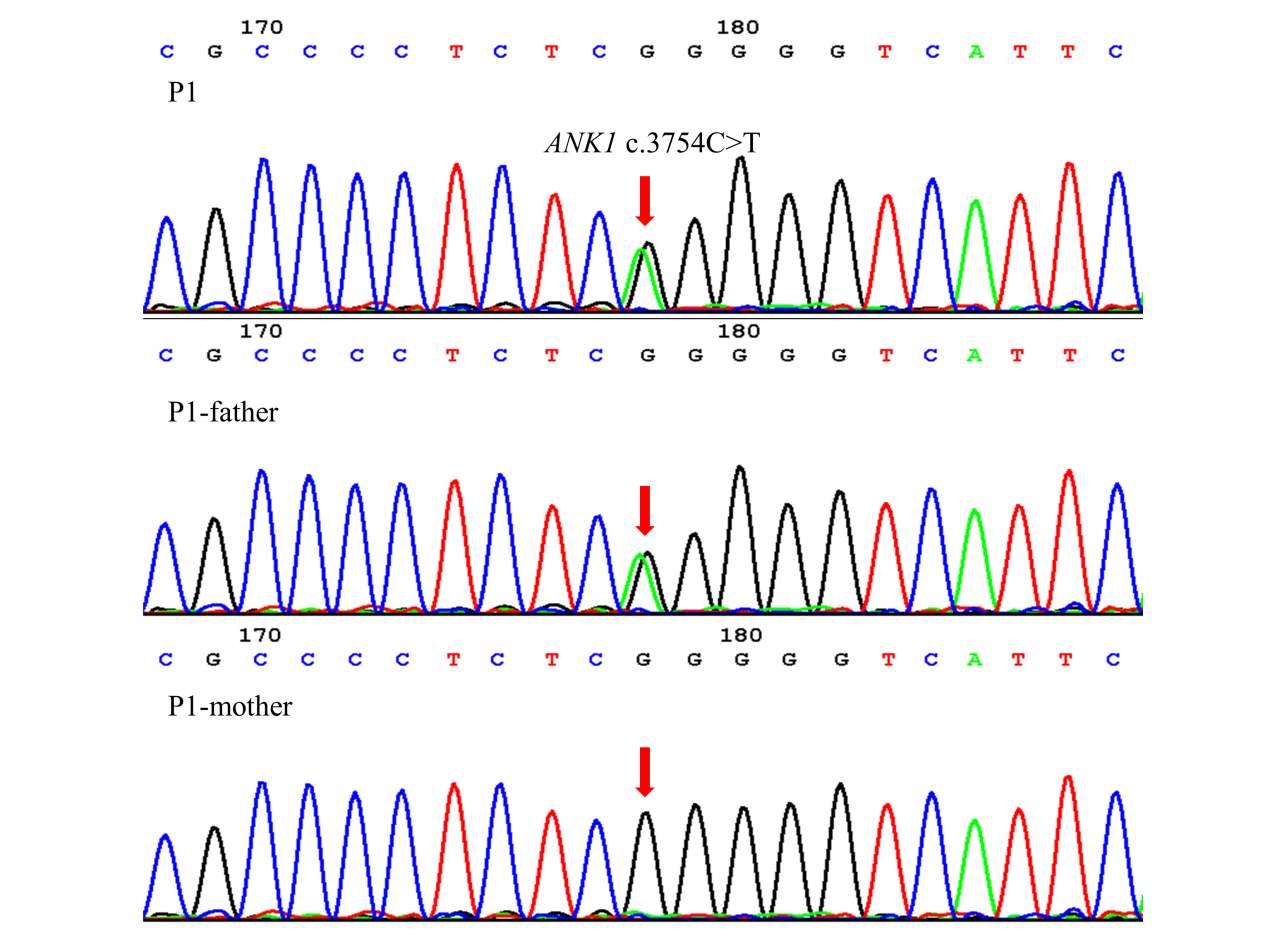


**P1** Sanger sequencing confirmed a heterozygous mutation of the *ANK1* gene (NM_020476.2) from the father in patient 1: c.3754C>T.

**P2** Sanger sequencing confirmed a heterozygous mutation of the *SPTB* gene (NM_001024858.2) from the father in patient 2: c.5266C>T.


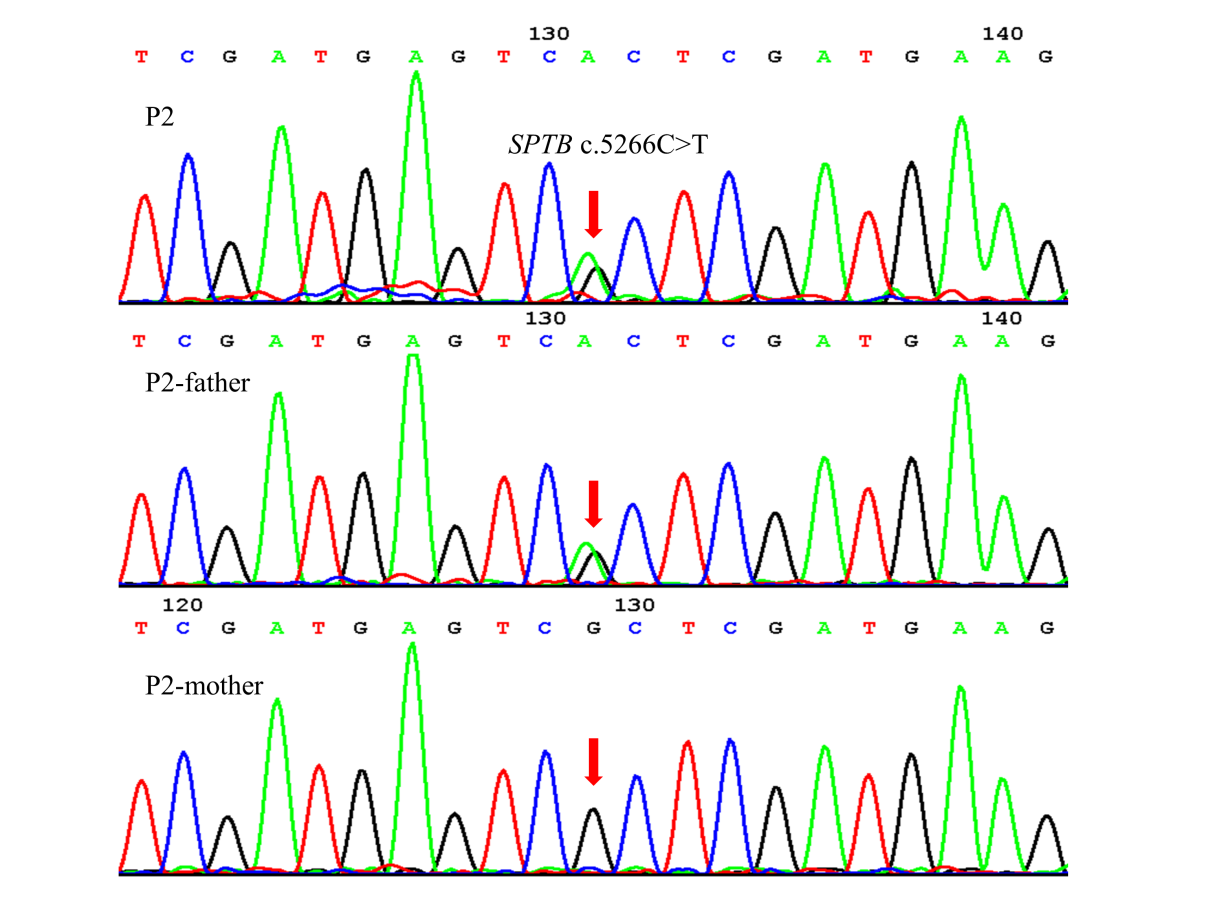


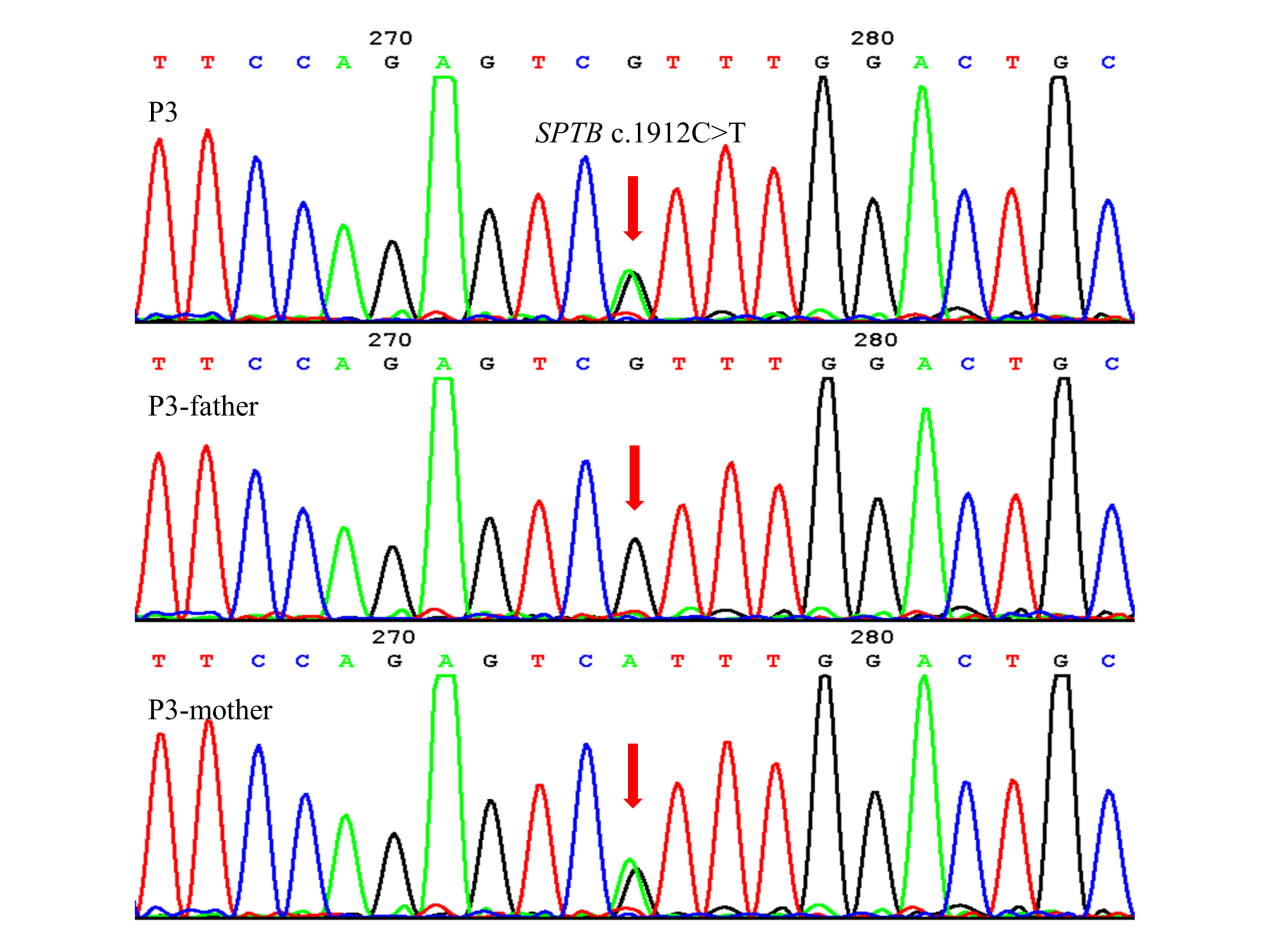


**P3** Sanger sequencing confirmed a heterozygous mutation of the *SPTB* gene (NM_001024858.2) from the mother in patient 3: c.1912C>T.


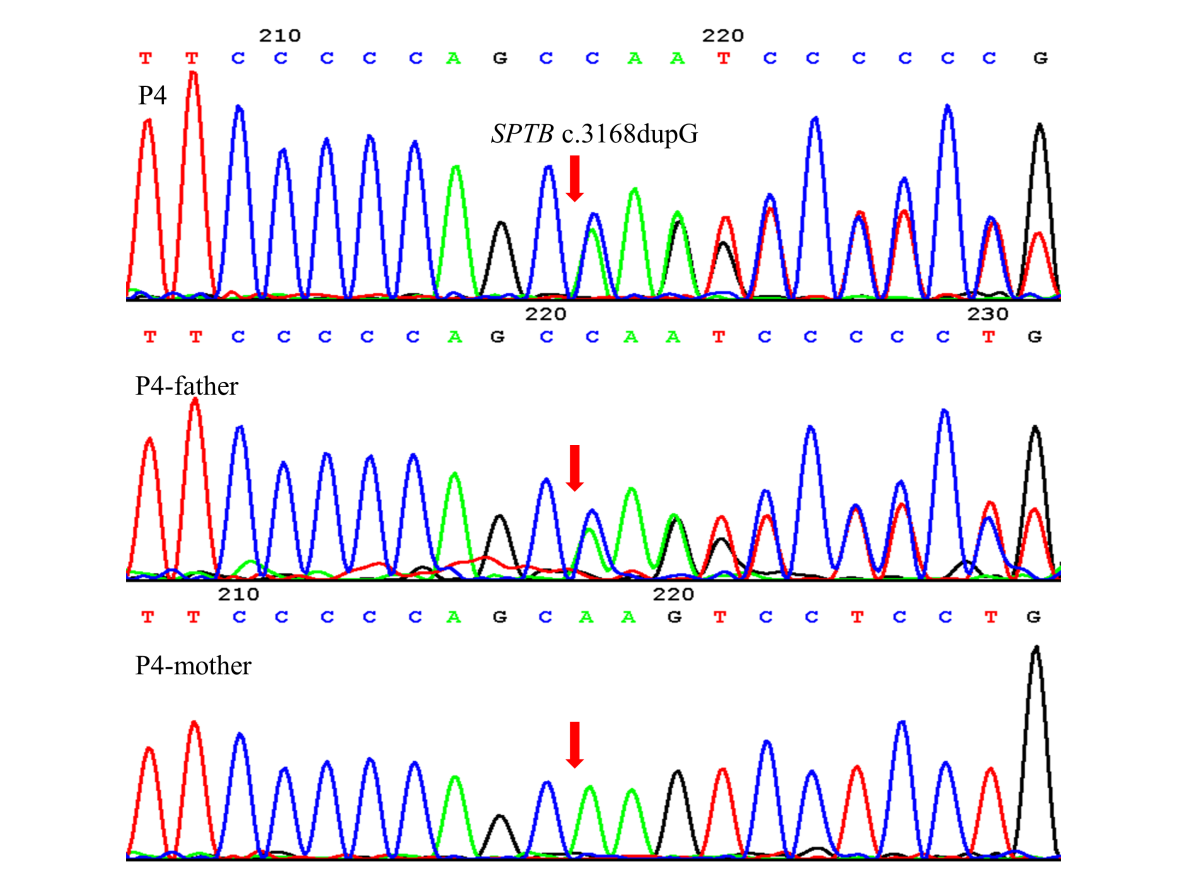


**P4** Sanger sequencing confirmed a heterozygous mutation of the *SPTB* gene (NM_001024858.2) from the father in patient 4: c.3168 dupG.


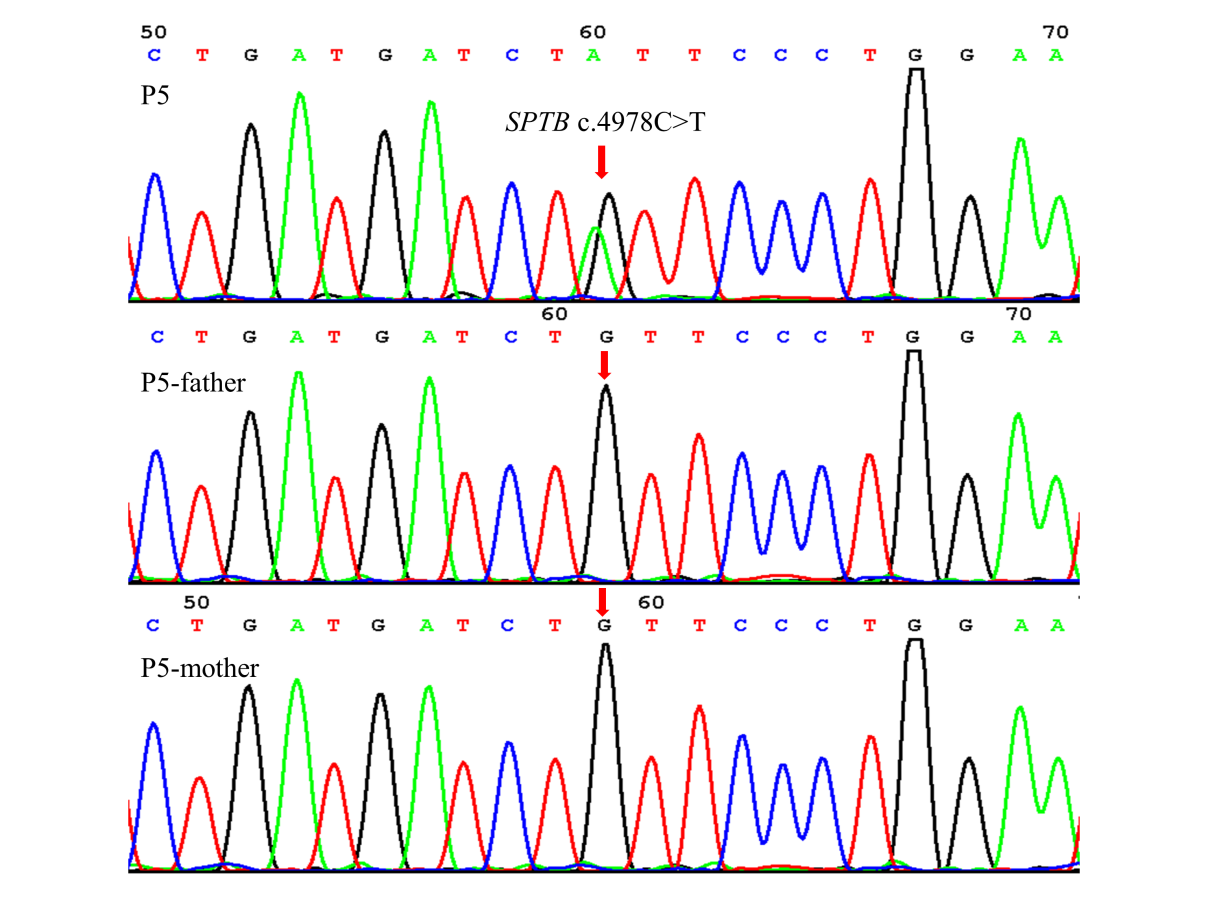


**P5** Sanger sequencing confirmed a *de novo* heterozygous mutation of the *SPTB* gene (NM_001024858.2) in patient 5: c.4978C>T.


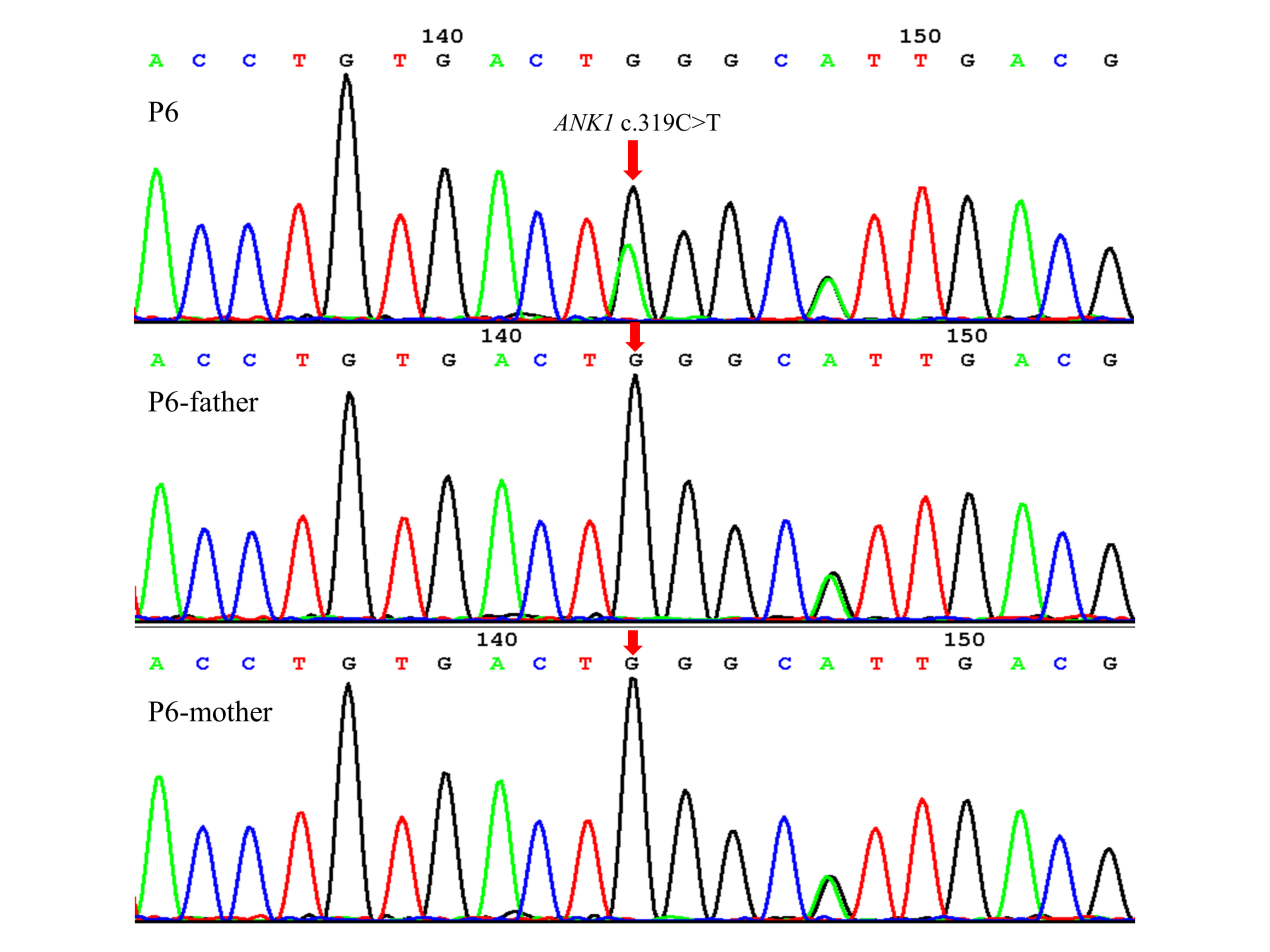


**P6** Sanger sequencing confirmed a *de novo* heterozygous mutation of the *ANK1* gene (NM_020476.2) in patient 6: c.319C>T.


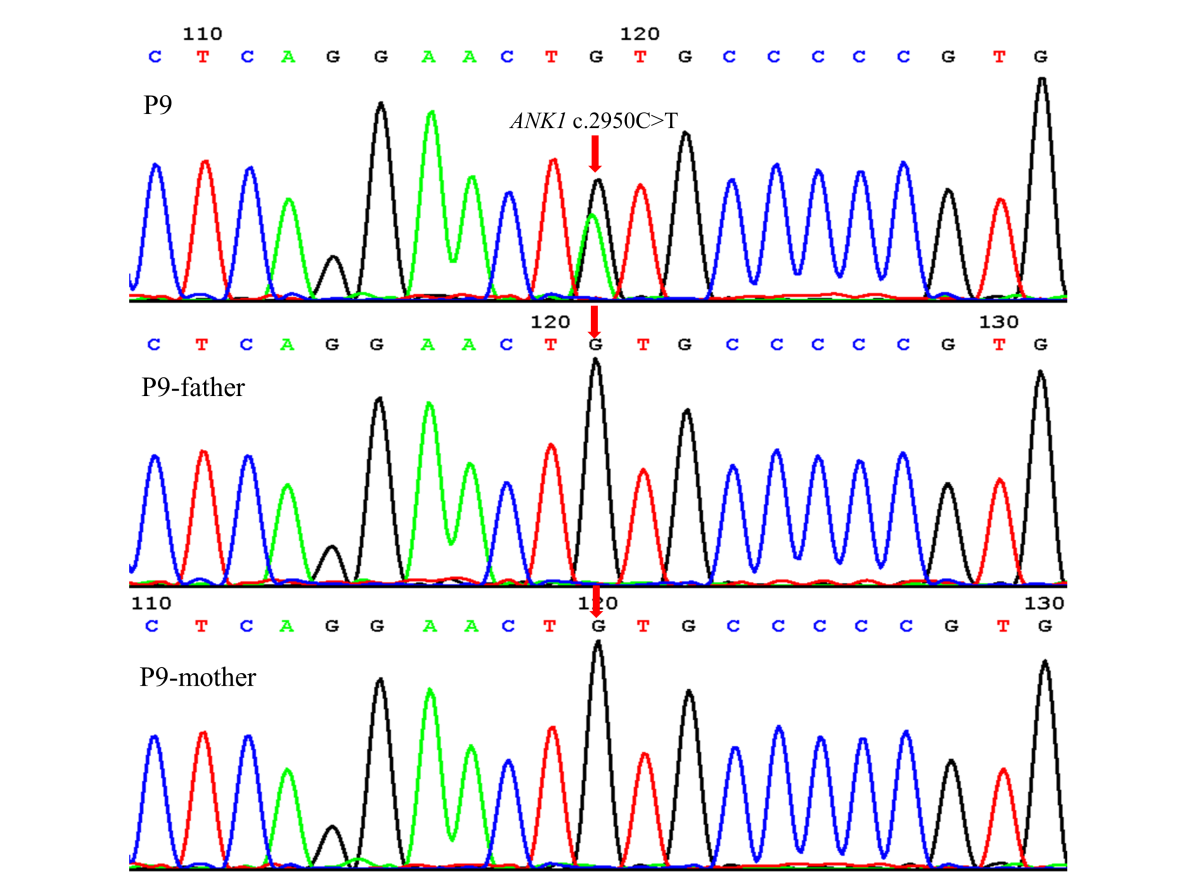


**P9** Sanger sequencing confirmed a *de novo* heterozygous mutation of the *ANK1* gene (NM_020476.2) in patient 9: c.2950C>T.


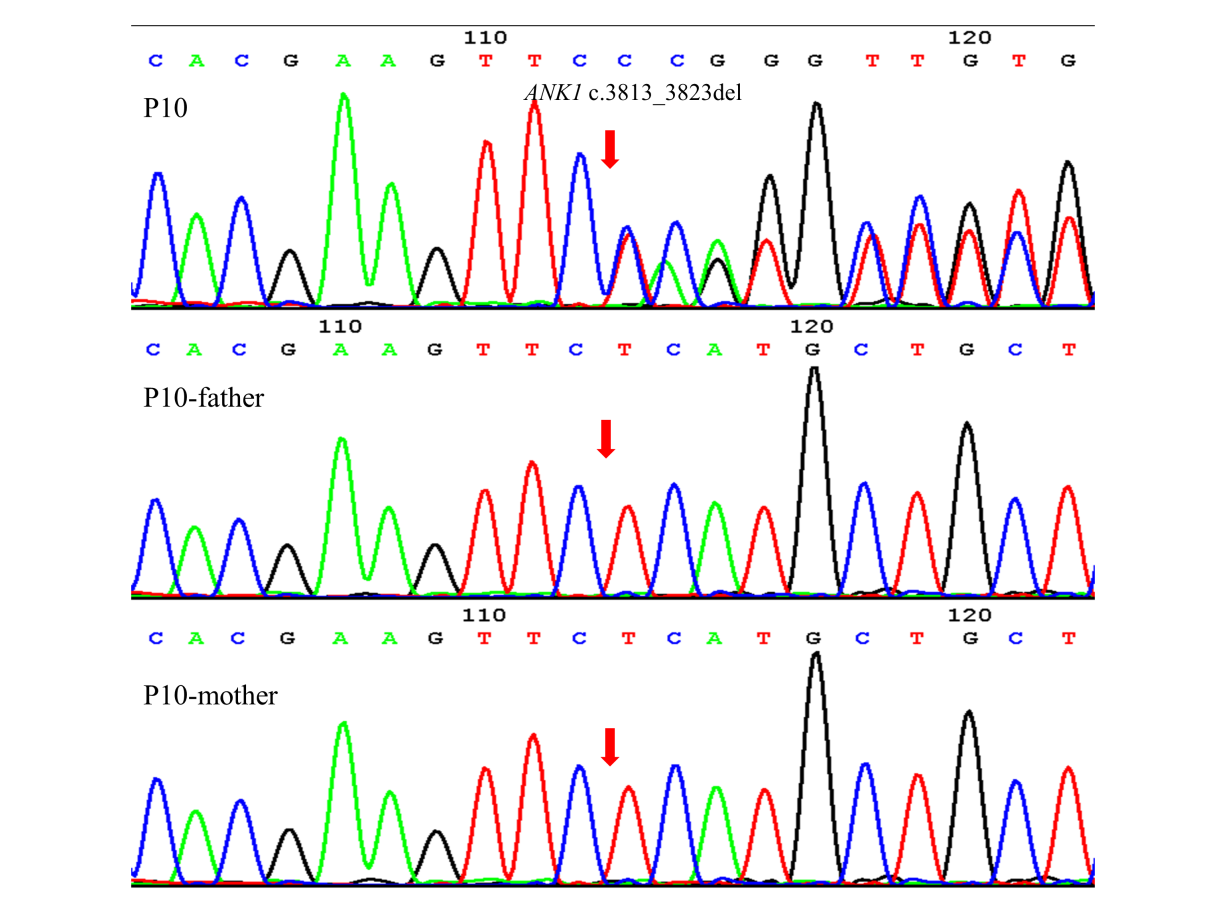


**P10** Sanger sequencing confirmed a *de novo* heterozygous mutation of the *ANK1* gene (NM_020476.2) in patient 10: c.3813_3823del.


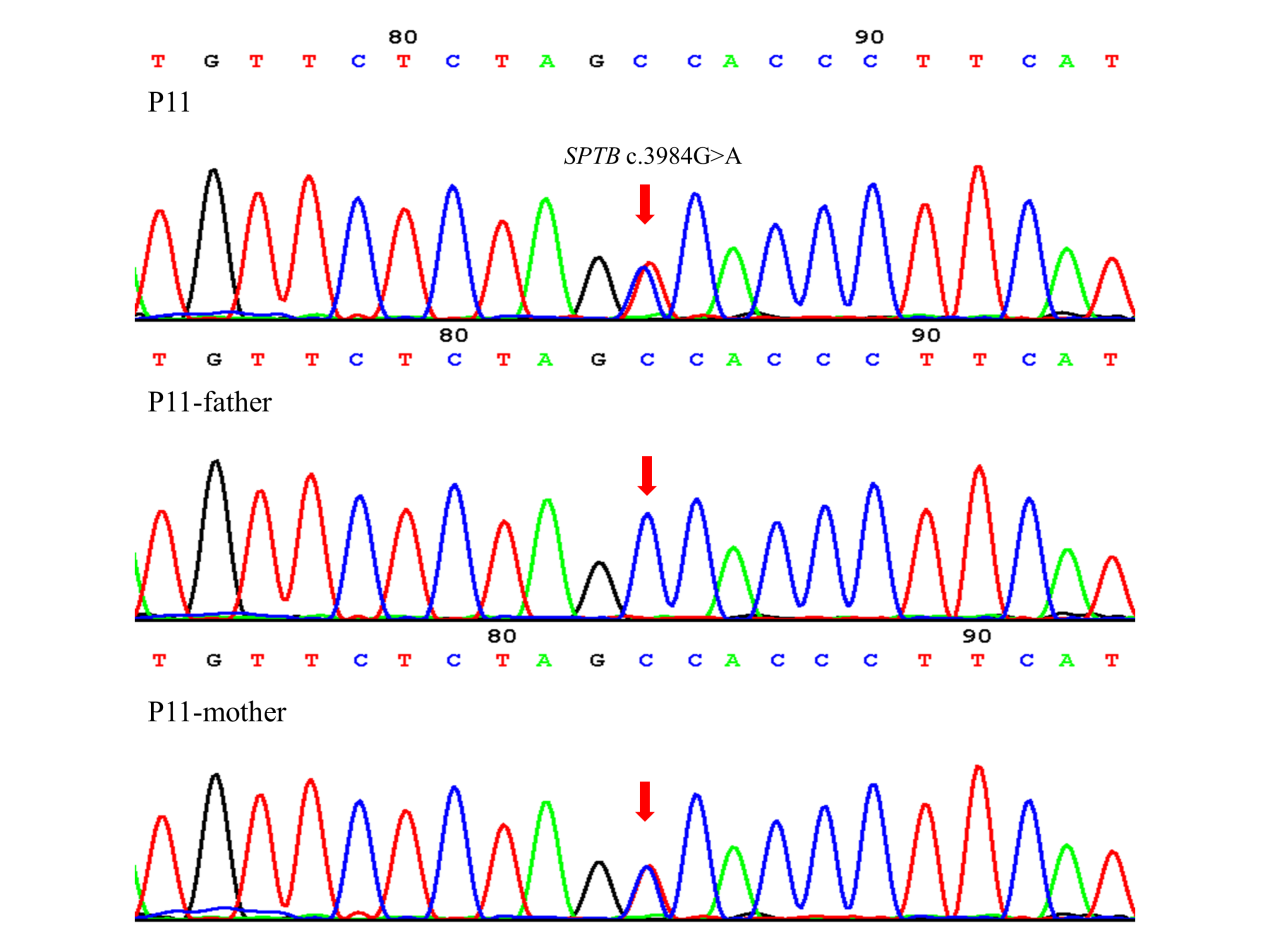


**P11** Sanger sequencing confirmed a heterozygous mutation of the *SPTB* gene (NM_001024858.2) from the mother in patient 11: c.3984G>A.


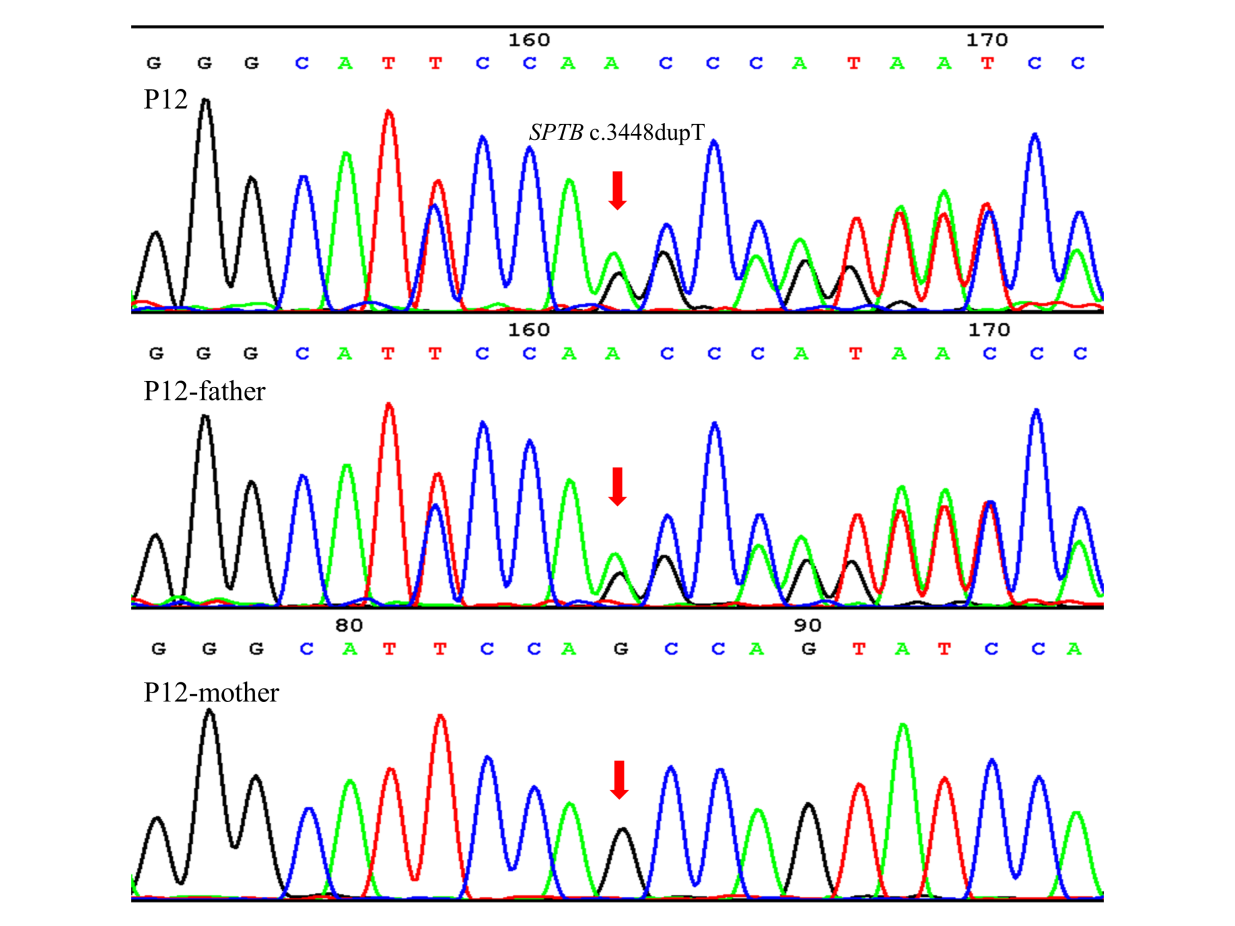


**P12** Sanger sequencing confirmed a heterozygous mutation of the *SPTB* gene (NM_001024858.2) from the mother in patient 12: c.3448dupT.


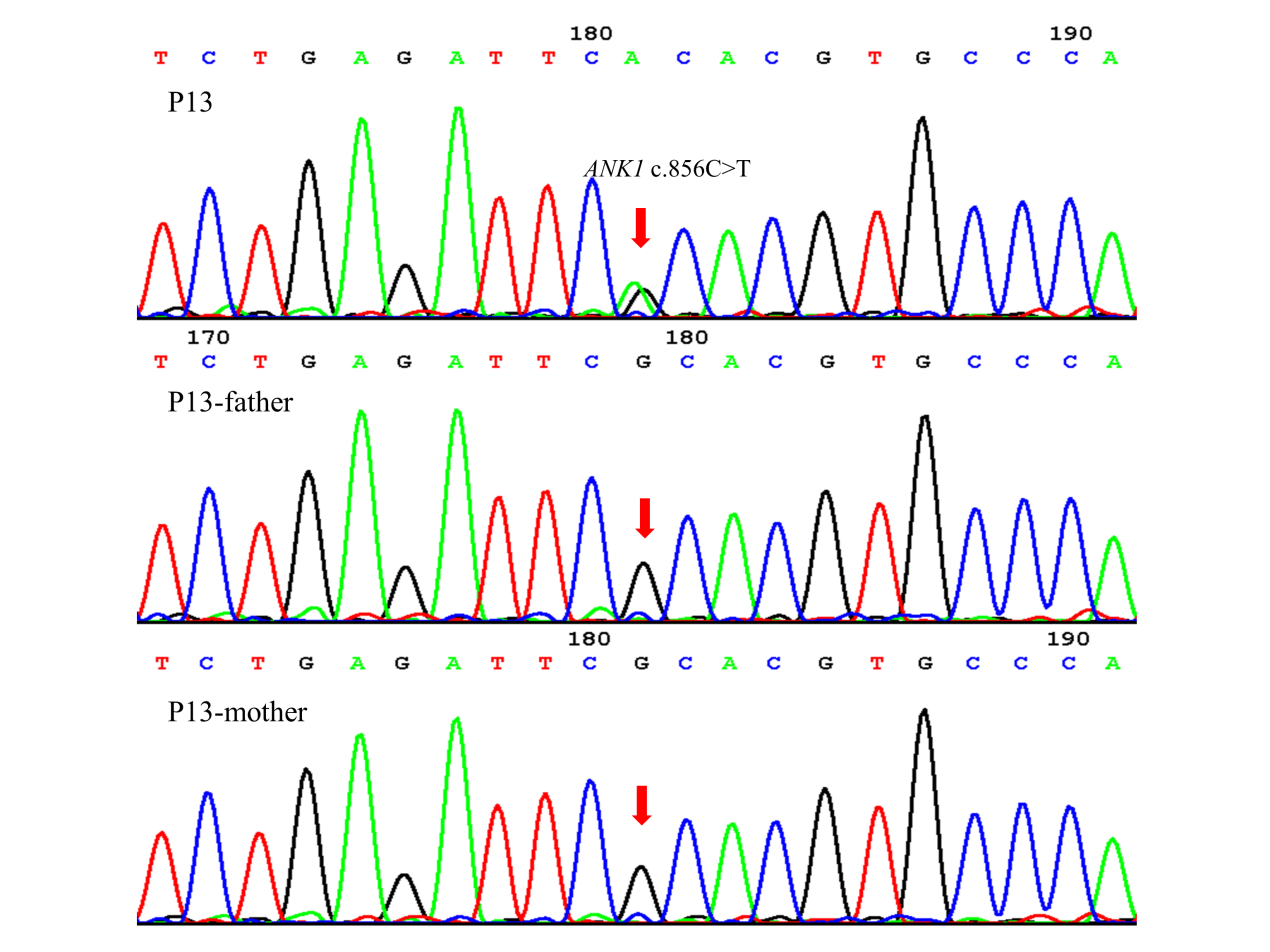


**P13** Sanger sequencing confirmed a *de novo* heterozygous mutation of the *ANK1* gene (NM_020476.2) in patient 13: c.856C>T.


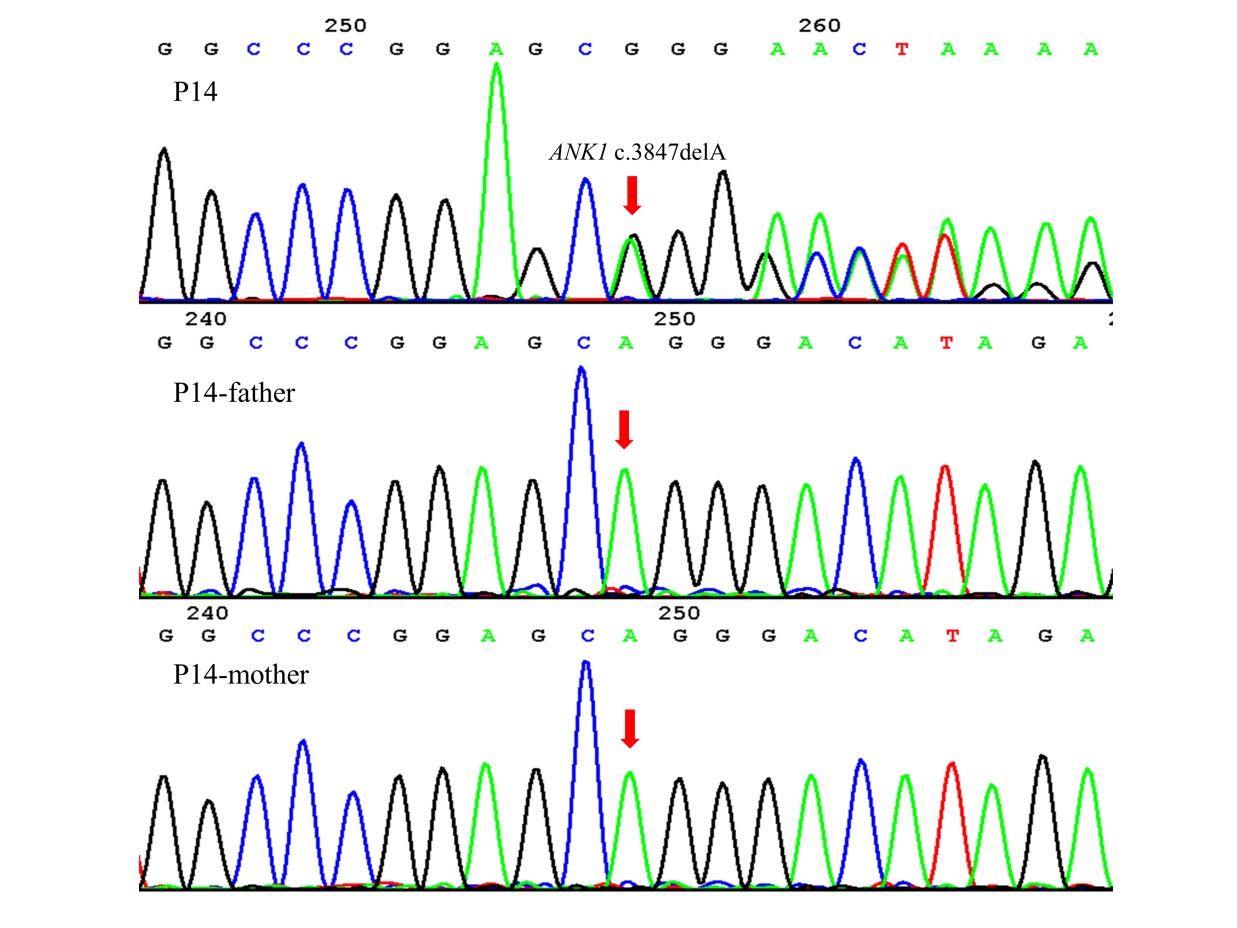


**P14** Sanger sequencing confirmed a *de novo* heterozygous mutation of the *ANK1* gene (NM_020476.2) in patient 14:c.3847delA.
